# Pros and Cons of Human Brain Organoids to Study Alzheimer’s Disease

**DOI:** 10.14336/AD.2024.1409

**Published:** 2025-12-03

**Authors:** Andrea Sainz, Fernando Pérez-Cerdá, Alberto Pérez-Samartín, Mitradas Panicker, Asier Ruiz, Carlos Matute

**Affiliations:** ^1^Achucarro Basque Center for Neuroscience, University of the Basque Country, CIBERNED and Biobizkaia, 48940-Leioa, Spain.; ^2^Department of Physiology & Biophysics, University of California, Irvine, California, USA.

**Keywords:** Alzheimer´s disease;, neurodegeneration, cerebral organoids, therapy

## Abstract

There is increasing pressure for researchers to reduce their reliance on animals, particularly in early-stage research. The main reason for that change arises from the different biological behavior of humans that leads to frequent failure of translating data from bench to bed. The advent of organoid technology ten years ago, along with the feasibility of obtaining brain organoids in most laboratories, has created considerable expectations not exempting frustration. In this review, we make a critical appraisal of the advantages and limitations of studying Alzheimer´s disease in brain cortical organoids derived from inducible pluripotent stem cells (iPSCs). While dealing with human neurons and glia in 3D poses a tremendous advantage versus murine brain cells, organoids typically lack microglia, blood vessels, immune interactions as well as proper CNS neuropil. In turn, they have relatively few oligodendrocytes and low myelination. In addition, lengthy procedures to get proper mature organoids constitute an additional limitation that may also affect the native biological properties of neurons and glia. We conclude that human brain organoids, while popular and useful, remain a model that needs further refinement before bringing substantial value to study Alzheimer´s disease.

## SEARCH AND SELECTION STRATEGY

The bibliographic search strategy was carried out in August 2024, using the following bioethical and biomedical databases: PubMed, Medline, Web of Science Core Collection, and Cochrane. To guide the search, we establish a series of tools or search terms in accordance with the agreed theme. Through an indexing process, the free terms were replaced by standardized and unambiguous terms (MeSH terms) targeting ‘Organoids’ and ‘Alzheimer's disease’. The logical relationship between terms was achieved using Boolean operators and truncations to retrieve those articles that had the same grammatical root in the databases that made the option possible. Articles in English were considered for full text analysis. We excluded abstracts for conferences and societies. All articles that meet our criteria are listed in the References section.

## 1. Brain organoids recreate the complex cellular diversity present in the human brain

Since Alzheimer´s disease (AD) is the most prevalent neurodegenerative disease of the Central Nervous System (CNS), there has been considerable effort in using neural organoid models to study this disorder. Organoids, in general, are the result of an innate ability of specific cell types to self-organize and emulate the fundamental functional properties and complex structure of various organs or tissues [1-5]. The result is a miniature in vitro version of a human organ, characterized by many similarities both in its architecture and in physiology, albeit with significant differences too, with respect to the original organ. Despite the valuable opportunities that animal models have provided for translational discoveries, it is important to recognize the intrinsic limitations stemming from differences in physiology compared to humans, which human organoids attempt to transcend. Earlier work using two-dimensional (2D) tissue or human and animal cell culture models have also provided novel insights into aspects of disease biology. However, these models tend to be static and simplified and have lack the ability to replicate the dynamics and complexity of biological processes in vivo. These restrictions have encouraged the urgent need to develop in vitro platforms that are more dynamic and deeply complex.

In this review, we will discuss the complex cellular interactions involved in AD as well as the main advantages and disadvantages in the use of three-dimensional (3D) human cell culture models taking into account the most recent advances in the creation and development of various neural organoid models.

## 2. The biology of neurons and glial cells and their complex roles in AD

AD is the most prevalent neurodegenerative disorder globally, with aging being the primary risk factor. It is characterized by a gradual loss of mnemonic and cognitive function because of the abnormal agglomeration of amyloid beta (Aβ) plaques and tau hyperphosphorylation-derived neurofibrillary tangles (NFTs), leading to neuronal demise and brain atrophy [6-9].

The finding of amyloid plaques isolated from individuals with AD and Down syndrome (DS) in the mid-1980s placed Aβ protein as a key event in disease physiopathology [10-12]. This connection fueled the conjecture that the gene that encodes the Aβ protein might reside on chromosome 21, given that full trisomy of chromosome 21 had been found to play a role in the genesis of DS. Likewise, DS brains display an early pathology of amyloid plaques, culminating in dementia in most cases around 50 years of age [13,14]. This hypothesis gained ground when it was revealed that the amyloid precursor protein (APP) gene, which encodes a larger type 1 transmembrane protein, from which the Aβ peptide is cleaved, was located on chromosome 21 [15,16]. These early milestones in AD research laid the foundation for the amyloid cascade hypothesis [17]. This hypothesis postulates that Aβ accumulation or deposition is the initial event in AD pathogenesis, triggering subsequent correlated pathophysiological events. These events include NFTs, synaptic dysfunction, inflammation, neurodegeneration, vascular aberrations, and finally dementia [17,18]. This predominant framework has driven research toward therapeutic strategies aimed at reducing Aβ production and aggregation. It has also inspired efforts to enhance Aβ clearance and ultimately decrease amyloid plaques. However, while the amyloid cascade hypothesis for AD has been pivotal in advancing research on the biological and pathological roles of Aβ, it has faced increasing criticism within the scientific field over the last decades [19]. On the other hand, the lack of success in clinical trials focusing on Aβ-targeted drugs has prompted the scientific community to explore alternative disease pathways and therapeutic approaches related to tau. Furthermore, the presence of NFTs is more closely related to clinical symptoms and neuronal loss [20,21].

In addition to Aβ and tau components, emerging evidence indicates that other neuronal stressors, such as neuroinflammation, play a role in the development of AD. The presence of reactive astrocytes and elevated levels of proinflammatory molecules occur throughout the progression of the disease. Astrocytes play a fundamental role in regulating neuronal connections by facilitating communication between pre and postsynaptic neurons [22]. They are considered essential components for promoting long-term potentiation (LTP), managing neural energy, and releasing signaling substances called gliotransmitters [23]. These cells also undergo changes in both their structure and function in response to diseases and abnormal conditions in the brain [24-26]. These changes trigger a response in astrocytes, the main purpose of which is to protect the brain from damage during the early stages of the disease. However, this response can have detrimental effects in more advanced stages of the disease [27,28]. In this regard, the beneficial properties of astrocytes are compromised as the gliosis reaction continues to evolve and progress. A prolonged state of astrocytic activation leads to an increase in the release of inflammatory cytokines and the promotion of an environment conducive to neuronal damage, while they lose their neuronal support and nutrition functions [29]. Notably, astrocytes derived from iPSCs of familial (FAD) and sporadic (SAD) AD patients display an autonomous pathological phenotype that includes atrophy suggesting a relevant role of astroglia in the development of AD [30].

Oligodendrocytes (OLs) also hold a genuine interest in the pathological context of AD, considering their role in the generation of myelin and support for axons [31]. Alterations in white matter (for example, lesions, volumetric decrease, microstructural detriment) and the demyelinating process have been widely reported in the context of the aforementioned pathological entity [32].

The research has also revealed significant data from whole exome sequencing studies, which have implicated the innate immune system as a critical element in the pathogenesis of AD. Recent discoveries have identified rare variants in the TREM2 gene (Triggering Receptor Expressed on Myeloid Cells 2), which is expressed in microglia. These variants increase the risk of developing AD by 2- to 4-fold, a risk level comparable to that conferred by the apolipoprotein E4 allele [33-35]. Thus, the expression of TREM2 in microglia appears to regulate the ability of these cells to engulf and eliminate cell debris, as well as their response to brain insults [36,37]. This highlights the role of TREM2 in modulating the neuroinflammatory processes that drive AD pathogenesis. Furthermore, ongoing accumulating evidence suggests that TREM2 activity is closely linked to lipid metabolism in microglia [38]. A recent analysis found that Trem2^-/-^ microglia in transgenic mice exhibit an inability to amplify transcripts associated with activation, lipid catabolism, and phagocytosis in response to myelin injury. This results in inefficient elimination of myelin residues, axonal dystrophy and long-lasting demyelination [39]. A number of AD susceptibility genes, apart from TREM2, also display preeminent or selective expression in microglia, e.g., CD33, INPP5D, MS4A6A and PLCG2. Therefore, it is plausible that they may influence the same activities and pathways regulated by TREM2 [40,41].

Finally, brain health depends on the vasculature of the brain, another fundamental element of great importance in this neurodegenerative disease [42]. The specialized features of blood vessels in the brain originate from a complex cellular community that interacts in a coordinated manner creating the blood-brain barrier (BBB). Among these cells are endothelial cells (ECs), which line the interior of the vessels; smooth muscle cells, surrounding the vessel; the pericytes, in close relationship with the basal membrane; immune cells near the vessels; and astrocytes surrounding the structure [43,44]. This cellular heterogeneity facilitates selective and hemodynamically sensitive transport of molecules between the bloodstream and the brain [45,46]. Consequently, BBB dysfunction could allow access of toxic molecules and cells from the blood, triggering an immune response related to neuroinflammation. This chain of events leads to degeneration of the neurovascular unit [47]. Likewise, the deterioration of the blood-cerebrospinal fluid (CSF) barrier, located in the choroid plexus (ChP) of the cerebral ventricles, due to the presence of Aβ, causes capillary damage and interstitial fibrosis, contributing to cerebral neurotoxicity [48]. In addition, alterations in the composition and production of CSF have been described. This is due to a reduction in the capacity of the ECs of the ChP to synthesize essential proteins required for CSF production. A decrease in the expression of ion transporters involved in its secretion has also been reported [49-52]. All of these dysregulations converge to accelerate neural network degeneration, nerve cell loss, and ultimately cognitive decline.

These intricate and dynamic interactions between cell varieties are factors that contribute to the progression of the disease. This underscores the need to explore a diversity of approaches that lead to a range of effective therapies for this neurodegenerative condition.

## 3. Neural divergences across species: A comparative perspective

Transcriptomic and epigenomic analysis methods at the single-cell level have proven effective in unraveling the cellular composition of complex brain tissues by examining gene expression patterns and underlying regulatory mechanisms.

Transcriptomic analysis of various regions particularly relevant for exploring cellular evolution in rodents and primates, has revealed a remarkably conserved cellular composition across the brain. However, despite this general conservation, species-specific adaptations are also evident, including differences in cell type proportions, gene expression, DNA methylation, and chromatin state [53]. Thus, the temporal cerebral cortex of individuals with AD shows a significant alteration in the expression of 100 genes in comparison to unaffected individuals. These genes have previously been identified as risk factors for AD through genome-wide association studies (GWAS). However, mice carrying a specific mutation in the APP gene showed 27 genes whose expression was significantly altered in the temporal cortex [54]. This divergence is especially notable in non-neuronal cells, given that human microglia express the vast majority of genes related to susceptibility to AD, while rodent microglia only express a fraction of these genes [55]. Along with microglia, brain ECs and pericytes exhibit the greatest transcriptional divergence [56,57]. Disparity in gene expression profiles is a result of evolution and may contribute to phenotypic differences between species. Indeed, human astrocytes exhibit larger size and more complex morphology compared to their mouse counterparts [58,59]. Likewise, differences have been discerned in astrocytic metabolism, susceptibility to oxidative stress, attributable to variations in mitochondrial physiology and detoxification pathways, and in molecular responses under inflammatory conditions between both species [60]. Molecular differences between OLs of different species have also been the subject of research. Proteomic analyzes of purified myelin reveal that there is a certain degree of divergence in the protein composition of human and mouse CNS myelin [61,62].

**Figure 1.**
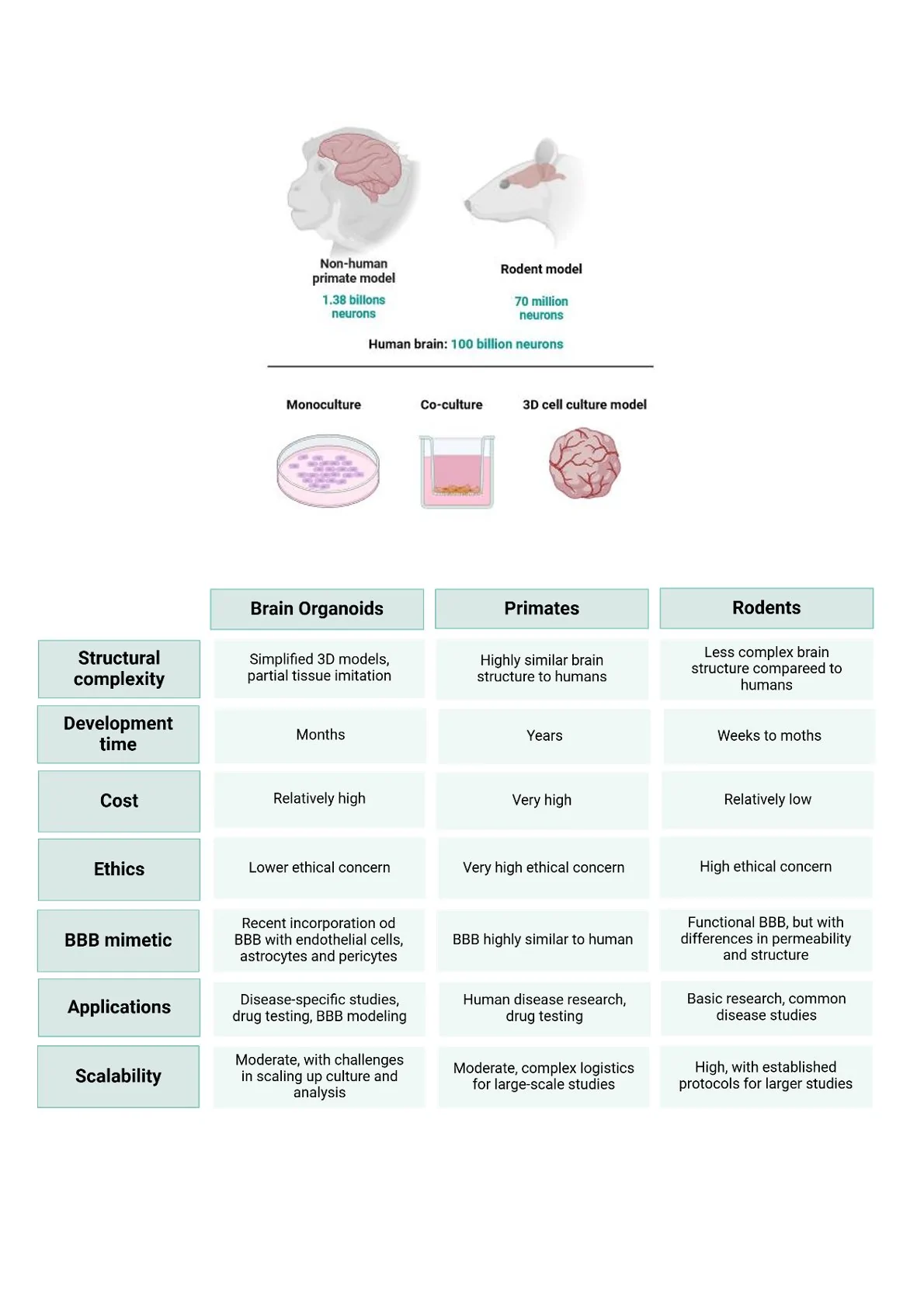
**Comparison of brain organoids and animal models: Overview of structural complexity, blood-brain barrier (BBB) mimicry, cost, ethics, applications and scalability [71-77]**. Created with BioRender.com.

Moreover, some primate species can spontaneously develop Aβ plaques and NFTs as they age [63-66]. Remarkably, the initiation and propagation of tau pathology in these primates closely mirror the patterns observed in AD patients. This finding strongly indicates that monkeys provide an unparalleled physiological model for investigating late-onset SAD. Environmental toxicants might contribute to AD pathogenesis, exemplified by the study conducted by Yang et al. [57], which utilized methanol administration to induce an AD model in monkeys. Furthermore, various research efforts have endeavored to create AD models through intracerebral or lateral ventricular injections of synthetic or patient-derived Aβ oligomers [67-70]. These approaches have led to early pathological manifestations, such as dendritic spine reduction, heightened inflammation, and synaptic dysfunction, as well as widespread Aβ accumulation and hyperphosphorylation of tau, the microtube-associated protein (P-tau) [67-69]. However, despite these findings, their use involves significant ethical concerns (Fig. 1) [58,71-77].

**Figure 2.**
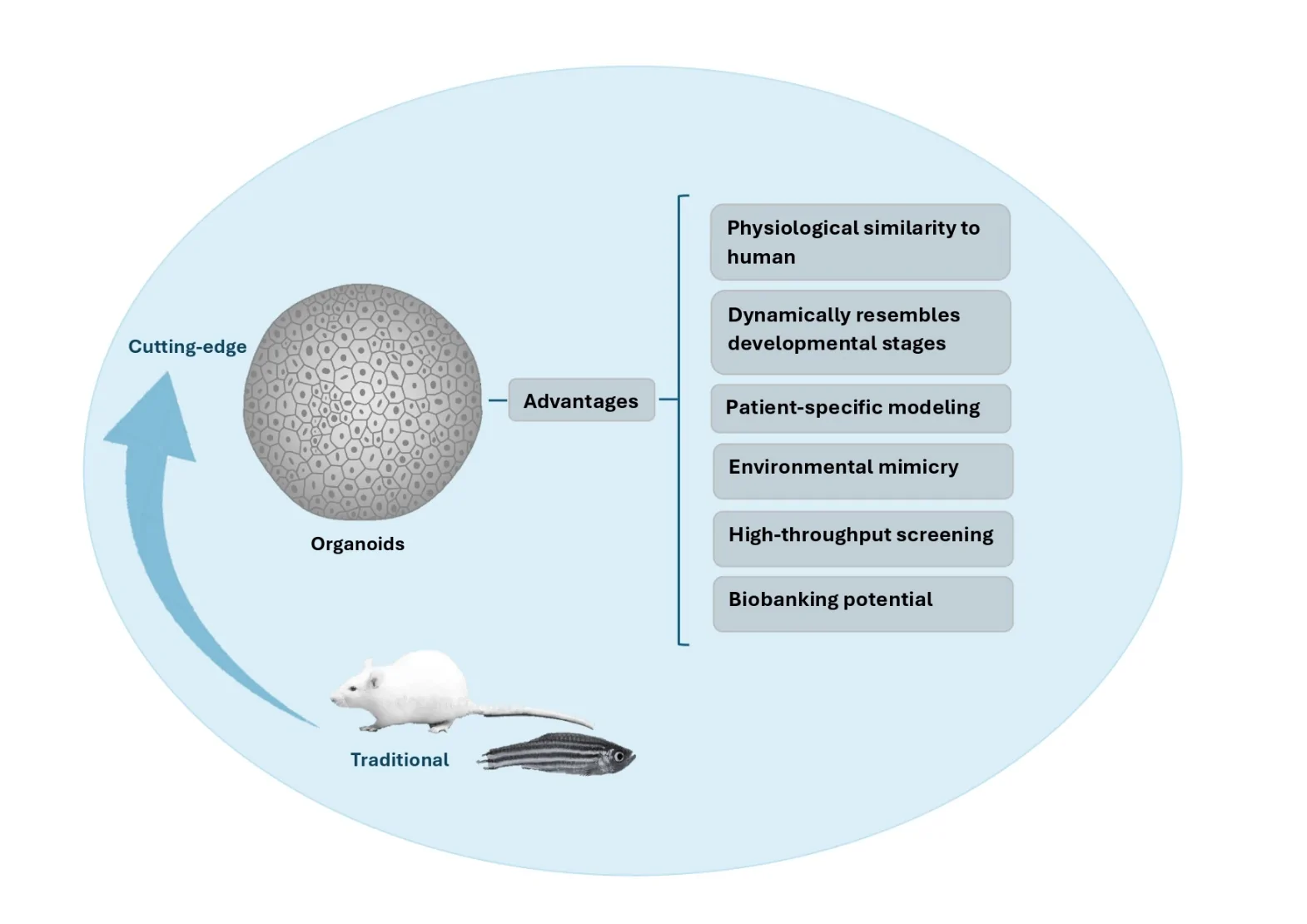
**Leveraging brain organoids: Pivotal advantages in modeling neurodegenerative diseases**. Brain organoids currently stand as the most sophisticated in vitro model for studying human brain development and disease, bridging the gap between basic science and clinical applications. Their ability to mimic the intricate cellular composition and physiological processes of the human brain allows for a more accurate representation of disease mechanisms, particularly for neurodegenerative conditions like AD and Parkinson disease (PD). Importantly, brain organoids recapitulate the complex process of human cortical development, including the characteristic organization of the progenitor zone with abundant external radial glial stem cells and the epigenomic signatures of the human fetal brain [78, 79]. Single-cell RNA sequencing analysis comparing brain organoids to the fetal neocortex has revealed that organoids in vitro closely mimic human neocortex development [80]. These findings strongly support the utility of human brain organoids in studies of human development. In comparison to traditional 2D cultures and animal models, organoids offer a more relevant and scalable platform for high-throughput drug screening, enabling the identification of potential therapeutic candidates in a human-like context [81]. The development of patient-specific organoids derived from iPSCs has further advanced the field, allowing researchers to explore disease variability at an individual level and opening the door to personalized medicine. Moreover, organoids are becoming integral components of biobanks, providing valuable resources for large-scale studies on disease progression, treatment efficacy, and cellular behavior [82]. Despite certain limitations, organoids remain a promising and versatile tool that overcomes many of the constraints posed by alternative models, significantly enhancing our ability to study human-specific neurobiology. Created using Microsoft.

These species-dependent divergences represent a barrier in translational research, as more than 90% of neurological drug candidates that show promising results in animal studies fail in human clinical trials. Organoids from patients with diseases reproduce the cellular phenotypes related to the pathology under study, providing the opportunity to elucidate the outcome of the disease in different tissues. In addition, they allow susceptibility testing to specific drugs and the creation of personalized therapeutic strategies (Fig. 2).

## 4. Organoid derivation methods and their limitations.

Pluripotent stem cells, namely, embryonic stem cells (ESCs) and induced pluripotent stem cells (iPSCs), have recently been explored for clinical use due to their remarkable ability to differentiate into cells of all three embryonic lineages [83]. The main concerns about ESCs involve ethical issues linked to the use of human embryos and the possibility of post-transplant immunological rejection. These conflicts have driven progress toward cellular reprogramming. In 2006, a team of scientists led by Shinya Yamanaka achieved a milestone by obtaining pluripotent stem cells from mouse fibroblasts [84]. Shortly after, in 2008, Yamanaka's team successfully replicated the same process using human cells [85]. The initial protocol used to obtain the first iPSCs from fibroblasts consisted of the combination of 4 transcription factors known as the “Yamanaka cocktail”, KLF4, SOX2, OCT4 and cMYC, encoded in a retrovirus. Over-expression of these factors had the ability to reverse the differentiation of somatic cells and reprogram them back into pluripotent stem cells [86]. However, the risk of tumorigenesis implied by the introduction of the c-Myc oncogene and its permanent integration was an obstacle to its clinical application [87]. To date, various groups have emphasized the potential of this strategy by optimizing the process to increase its efficiency by excluding this transcription factor [88,89]. The establishment of other messenger RNA-based, episomal vectors, transient viral infections or protein reprogramming methods has also been considered for the generation of human iPSCs without permanent genetic alterations [90]. Differentiated iPSCs routinely achieve gene expression profiles that directly match various brain cell types present in the appropriate in vivo environment. Of particular significance, 2D cultures derived from iPSCs have emerged as indispensable tools for elucidating intricate cellular behaviors and pathophysiological mechanisms within a controlled experimental framework. These 2D systems allow for precise manipulation of the extracellular environment, facilitating the study of neuronal differentiation, synaptic formation, and the progression of neurodegenerative diseases [91]. IPSC-derived neurons cultured in 2D have been successfully employed to model AD, revealing key insights into Aβ plaque formation and tau protein aggregation [92-99]. Nevertheless, 2D monolayer platforms are inherently limited in their capacity to replicate the complex three-dimensional architecture of the brain. They lack the tissue-specific spatial and morphological cellular organization, essential cell-cell and cell-extracellular matrix (ECM) interactions, as well as the inclusion of immune system cells and the dynamics of waste accumulation and clearance, which are crucial for accurately modeling physiological and pathological processes [100,101].

In recent years, various attempts have been made to model specific brain structures more faithfully by employing organoids, which represent a significant advancement over traditional 2D cultures. A crucial question in optimizing organoid creation methods is whether to deliberately promote cell differentiation toward specific fates. This can be addressed by the presence or absence of exogenous morphogens and signaling molecules in the stem cell medium. Protocols that promote spontaneous differentiation by avoiding the addition of external factors to the environment lead to the generation of heterogeneous cell populations. These populations correspond to various areas of the brain, spanning from the retina to the hindbrain [102].

The research group led by Sasai pioneered the generation of brain organoids, using methods that guided differentiation towards the forebrain and protocols to obtain optic cups [103]. They were based on the cultivation of stem cell aggregates under serum-free conditions. This 3D aggregation system, which they named SFEBq (serum-free floating culture of embryoid body-like aggregates with rapid reaggregation), enabled the self-organized formation of mouse and human embryonic stem cell aggregates [104]. This was achieved by the addition of a molecule that generated gradients of intra-aggregate morphogens, such as the treatment aimed at promoting the rostral pallial region through FGF8 [103,104]. Thus, guided differentiation protocols encompass an initial phase of stem cell aggregation, a general neural induction stage, and, in numerous cases, short periods of exposure to specific morphogens to direct the formation of particular tissue patterns followed by a prolonged period of stimulation with growth factors.

**Figure 3.**
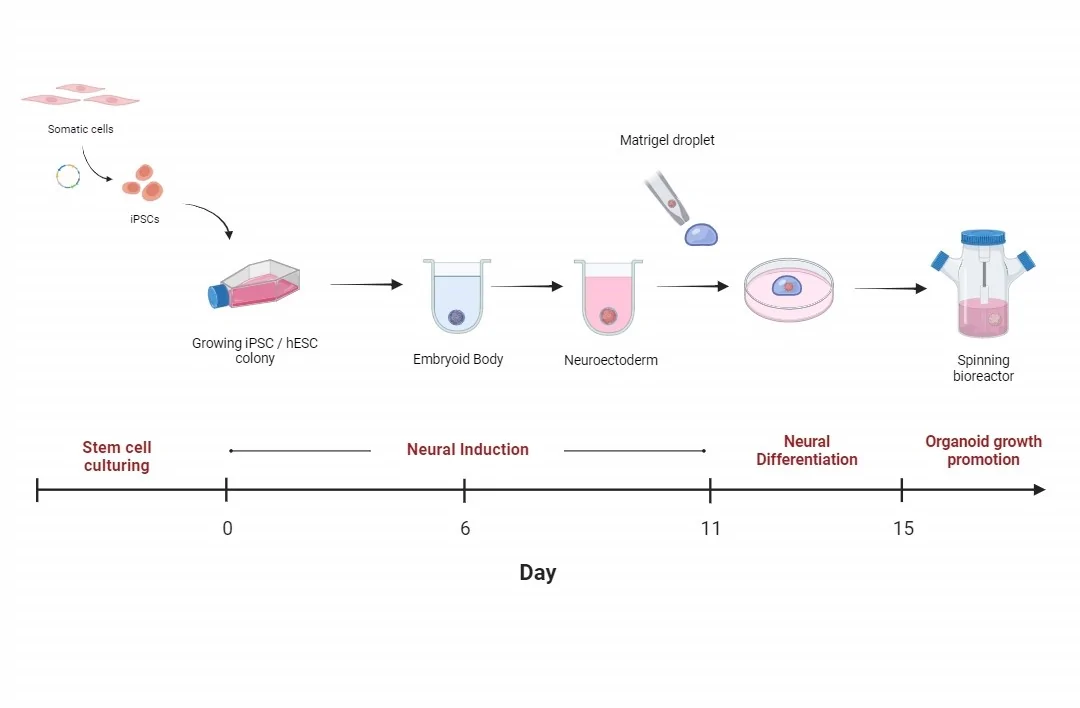
**Schematic illustration of the standard organoid formation process**. Overview protocol to generate brain organoids with defined differentiated compartments based on Lancaster et al. 2013 [79]. The process begins by seeding 4,500 iPSCs into round-bottom ultra-low-adhesion 96-well plates, creating spherical structures called embryoid bodies (EBs) in a culture medium appropriate for growth. After 6 days, EBs are transferred to low-adherence 24-well plates in neural induction medium for the next 5 days giving rise to neuroectodermal tissue. The individual tissues are then encapsulated in separate Matrigel droplets. Once the Matrigel is solidifies, tissue in droplets is supplemented with differentiation medium and maintained in stationary culture conditions for 4 days. Subsequently, Matrigel droplets containing the tissues are placed in a rotating bioreactor along with a differentiation medium designed to promote long-term viability of the organoids. This configuration facilitates the diffusion of oxygen, nutrients, and waste, thus contributing to the continued health and development of the organoids. The tissues are then transferred individually to separate Matrigel droplets. Created with BioRender.com

Lancaster et al. made significant strides in the field with their groundbreaking work on cerebral organoids [79]. These early efforts led to the development of organoids exhibiting various regional identities, organized as distinct domains capable of influencing one another (Fig. 3). In their seminal work, Lancaster et al. pioneered the development of brain organoids with a forebrain-like architecture by employing poly(lactide-co-glycolide) (PLGA) copolymer fiber microfilaments as a floating scaffold for the generation of elongated embryoid bodies (EBs). This is because most brain organoid culture procedures start from spherical structures or EBs, where the developing nervous tissue grows on the periphery of the EB. Lancaster and colleagues hypothesized that the variability in neural ectoderm formation could arise due to the unfavorable relationship between surface area and volume. Analysis using EB staining for specific markers of the germ layers, in early stages, revealed the generation of a more uniform and reproducible polarized neuroepithelium with reduced amounts of endodermal and mesodermal tissue. This advancement demonstrated a consistent ability for developing nervous tissue without compromising the organoid´s self-organization, leading to improved neuronal induction of the forebrain [105]. Despite these advances, undirected differentiation remains a challenge, with some cells adopting non-ectodermal fates. Consequently, ongoing research focuses on refining protocols to optimize the application of external signals to direct differentiation towards neurons [78,106]. The establishment of the foundations of the field paved the way for other scientific teams to develop organoids with specific regional destinations (spinal cord, basal ganglia, hippocampus, cerebellum, etc.) [107-110]. One of the challenges in the field of organoid research lies in the use of ECMs derived from animals, such as Matrigel used in the initial protocol. Matrigel is derived from the ECM secreted by Engelbreth-Holm-Swarm mouse sarcoma cells and is enriched in ECM proteins [111]. Despite its versatility and affordable cost, Matrigel is extremely complex. The undefined nature of this matrix can complicate the identification of signals essential for organoid structure and function, compounded by batch-to-batch variations of this material [112]. Aspects such as the arrangement of pores in the scaffolds, exposed surface area and porosity also play a crucial role in the relationship between cells and matrix. These mechanical properties of Matrigel samples exhibit notable heterogeneity [113]. Additionally, animal-derived cellular matrices raise ethical and clinical concerns in the context of eventual transplantation of human organoids due to their immunogenic potential [114]. The various specific applications of Matrigel-based organoids have been discussed exhaustively [115,116]. In view of these limitations, some of the protocols have dispensed with Matrigel altogether. Alternative methods for culturing Matrigel-independent organoids have also emerged. These include the use of ECMs derived from decellularized tissues and collagen, the application of synthetic polymer-based hydrogels and the incorporation of engineered ECM proteins [117-121].

### 4.1 Pluripotent Stem Cells used to model AD

Human AD brain organoids have been obtained from iPSCs that harbor genetic mutations found in FAD or the risk factor for the onset of SAD, the APOE4 allele. In the former, iPSCs derived from patients with genomic mutations in the PSEN1, PSEN2 or APP genes. In particular, 3D neural cultures derived from iPSCs with the PSEN1 E9 mutation or the APP K670N/M671L variant more faithfully recapitulate AD pathology including early amyloid plaque formation and synaptic dysfunction [122]. In turn, SAD patients carrying the APOE4 variant have alterations in lipid metabolism, impaired Aβ clearance and tau protein dynamics that exacerbates the formation of Aβ plaques and P-tau, reflecting the complex interplay between genetic risk factors and AD pathology [123-125].

**Figure 4.**
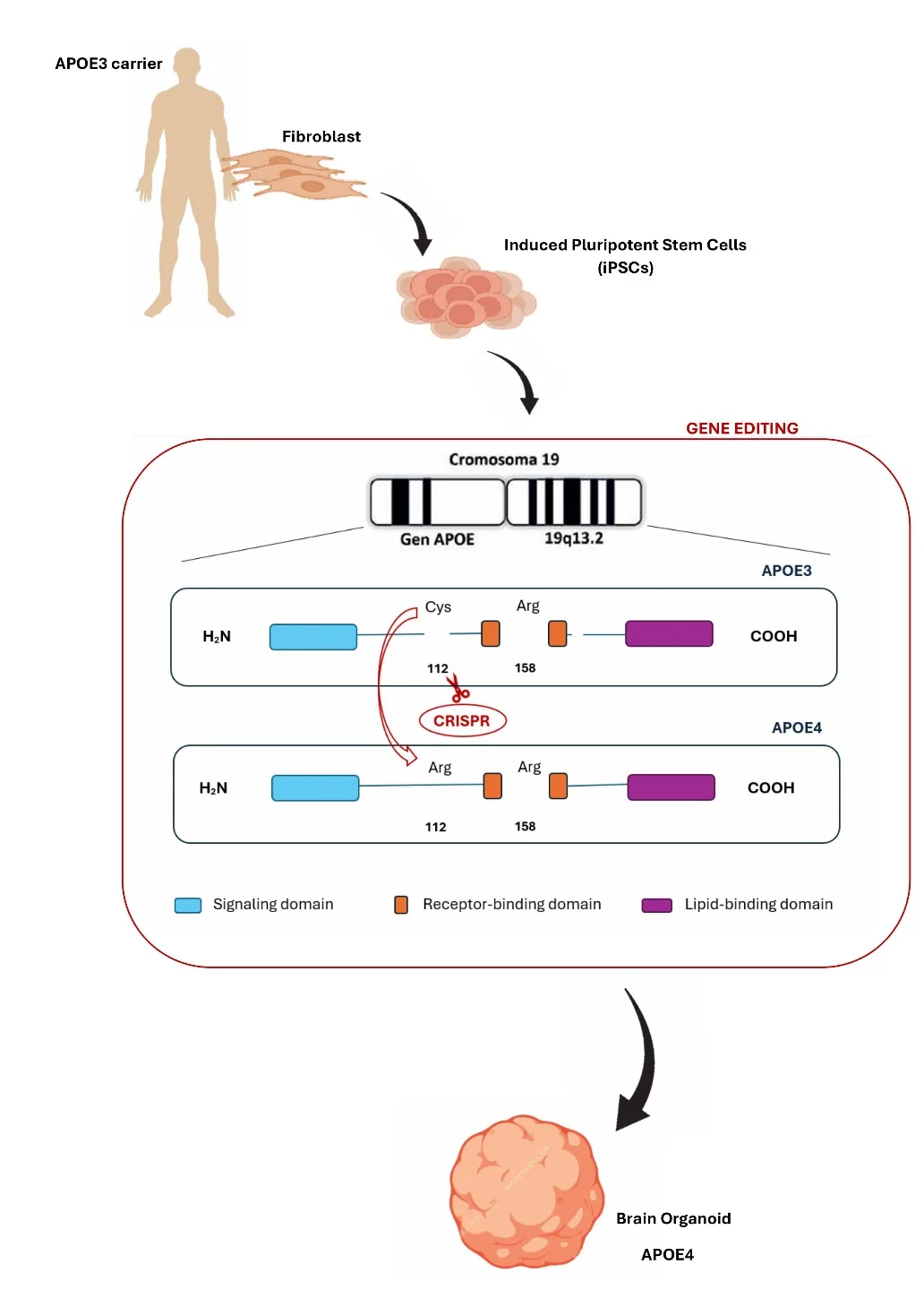
**Generation of isogenic APOE4 cerebral organoids for Alzheimer’s Disease modeling and drug screening**. Fibroblasts from a patient with the APOE3 allele are genetically engineered using CRISPR/Cas9 technology to convert the sequence into APOE4, a variant associated with an increased risk of AD. A plasmid vector containing a specific guide RNA and a repair template encoding the Arg112 mutation is used to induce homology-directed repair after Cas9-mediated cleavage. The edited cells are reprogrammed into iPSCs and differentiated into cerebral organoids for comparative analysis of the phenotypic effects between APOE3 and APOE4 variants. These isogenic cell lines can be employed to investigate the differential accumulation of amyloid-beta and tau proteins, assess neuroinflammatory responses, and study synaptic dysfunction associated with AD. Organoids from these cell lines provide a platform for high-throughput drug screening to identify compounds that may counteract the detrimental effects of the APOE4 allele, and for exploring gene therapy strategies aimed at correcting or mitigating the impact of APOE4 in a patient-specific context. Moreover, these models can contribute to understanding how APOE4 influences the blood-brain barrier integrity and lipid metabolism, providing insights into the systemic aspects of AD pathology. Created with BioRender.com

Moreover, the production and distribution of APOE in the CNS are not uniform across different cell types, which can have important implications for organoid modeling. Astrocytic APOE constitutes the major fraction of brain APOE, accounting for approximately 75-80% of total levels and is predominantly secreted into the extracellular space. In contrast, neuronal APOE, which accounts for about 15-20% of brain APOE, remains largely intracellular [126-128]. The differential contribution of these cell types to overall APOE levels and their secretion patterns is crucial for accurately modeling APOE-related pathology in organoids. To ensure the presence of astrocytes in organoids, it is important to note that mature astrocytes typically emerge after approximately 2.5 to 3 months of development, as indicated by the expression of markers such as GFAP (glial fibrillary acidic protein) and S100β (S100 calcium-binding protein-β) [129]. As previously mentioned, these astrocytes play a critical role in the overall APOE production, significantly contributing to the extracellular levels of APOE in the CNS. Understanding these dynamics is essential for guiding the design of organoids that more accurately represent the physiological and pathological roles of APOE, including how its dysregulation might contribute to AD. This consideration is particularly relevant when employing gene-editing techniques, such as CRISPR/Cas9, to introduce specific mutations or manipulate APOE expression in organoid models (Fig. 4).

In human AD, tau hyperphosphorylation is a multifaceted process influenced by a range of factors, including genetic predisposition and complex interactions between tau and Aβ [130]. Under physiological conditions, tau undergoes cycles of phosphorylation and dephosphorylation, regulated by kinases and phosphatases that maintain its function. In contrast, in AD, an imbalance occurs, leading to excessive proline-directed phosphorylation driven by kinases such as cdk5, GSK3, and calcium calmodulin kinase II, which disrupts tau function and promotes aggregation. Furthermore, proteins responsible for dephosphorylation, such as phosphatase 2, fail to maintain the balance, resulting in tau hyperphosphorylation [131,132]. Tau can be phosphorylated at various sites, with each site potentially affecting its aggregation properties differently, depending on the specific amino acid modified [133]. It should be noted that, while phosphorylation promotes tau aggregation, it is not strictly required for it. Modeling tau pathology in iPSC-derived brain organoids is challenging due to this complexity. Although these organoids offer a promising platform for AD studies, they often fall short in accurately replicating the diverse tau pathology observed in human brains. Specifically, these organoids frequently lack the full spectrum of tau isoforms present in human neurons, which affects tau oligomerization and fibrillization properties. The absence of these isoforms can lead to discrepancies in the formation and characteristics of tau aggregates compared to those seen in vivo [134].

**Table 1:** Summary of studies that have developed Alzheimer’s disease (AD) models using cerebral organoids derived from patient induced pluripotent stem cells (iPSCs).

FAMILIAL ALZHEIMER´S DISEASE (FAD) MUTATION		
PUBLICATION	IPSC CULTURING	PHENOTYPE
Raja et al. 2016 [143]	iPSCs from FAD patients (APP duplication; PSEN1 mutation); iPSCs of patients with duplication in the APP gene and mutation in the PSEN1 gene.	Robust and relevant phenotypes similar to AD. Large numbers of intra- and extracellular Aβ aggregates and significant P-tau immunoreactivity.
González et al. 2018 [144]	IPSCs of patients with duplication in the APP gene and mutation in PSEN1.	Aβ and P-tau aggregates, with a degree of cellular apoptosis proportional to the accumulation of protein aggregates. Over time, structures resembling extracellular senile amyloid plaques and intracellular NFTs associated with late-onset AD.
Ghatak et al. 2019 [145]	iPSCs from AD patients with APP Swedish mutation. iPSCs from AD patients with PSEN1 mutation.	Enhanced neuronal hyperexcitability; increased spontaneous firing rates; decreased neurite length; altered synaptic density, and aberrant calcium dynamics.
Kuehner et al. 2021 [146]	FAD patient-derived iPSC lines: PSEN1 ^Y155H^; PSEN1 ^M139V^; PSEN1 intron4 delection and APP ^V717I^	Aberrant 5-hydroxymethylcytosine (5hmC) regulation and altered DNA methylation patterns.
Yin et al. 2021 [147]	IPSCs from AD patients with PSEN2^N141I^ mutation.	Higher Aβ42/Aβ40 ratio, asynchronous calcium transient and enhanced neuronal hyperactivity.
Vanova et al. 2023 [148]	iPSC lines from AD patients with PSEN1 (A246E) and PSEN2 (N141I) mutations.	Defective tissue patterning and altered development; irregular layering and disrupted cytoarchitecture; delayed neuronal, and glial cell maturation.
Holubiec et al. 2023 [149]	iPSCs from patient-derived fibroblasts carrying the Swedish mutation in APP (APP^swe^).	Increased oxidative stress; elevated ROS production; mitochondrial membrane damage; dysfunction in mitochondrial respiratory chain, and ATP production.
SPORADIC ALZHEIMER´S DISEASE (SAD) MUTATION		
PUBLICATION	IPSC CULTURING	PHENOTYPE
Lin et al. 2018 [150]	Isogenic iPSC lines harboring homozygous APOE4 alleles.	Aβ aggregates and tau hyperphosphorylation; synaptic deficits; lipid accumulation, and disruptions in cellular signaling pathways associated with AD phenotypes.
Zhao et al. 2020 [151]	IPSCs derived from AD patients carrying APOE ε4/ε4.	Aβ aggregates; phosphorylated tau; synapse loss, and apoptosis.
Zhao et al. 2020 [152]	iPSCs derived from patients with APOE4 genotype.	Disrupted lipid homeostasis and greater accumulation of α-synuclein aggregates.
Park et al. 2021 [153]	iPSCs from patients with APOE4 mutation.	Aβ40/42ratio ; tau accumulation ; P-tau, and calcium transients.
Hernández et al. 2021 [154]	iPSCs from individuals with an APOE ε4/ε4 genotype.	APOE levels; Aβ40/42 ratio, and P-tau.
ALTERNATIVE PROTOCOLS		
PUBLICATION	IPSC CULTURING	PHENOTYPE
Pavoni et al. 2018 [137]	Primary fibroblast obtained from ATCC (CRL-2522) were reprogrammed into iPSC. No specific mutations. Small-molecule induction of AB-42 production.	Accumulation of Aβ-42 peptide; plaque-like structures, and neuronal dysfunction.
Alic et al. 2020 [141]	iPSCs derived from patients with Trisomy 21 (DS).	Increased Aβ-42; P-tau; amyloid plaques; BACE2 as a gene dose-sensitive suppressor; neurogenesis defects and altered brain development.
Chen et al. 2021 [142]	iPSCs derived from individuals without known familial genetic mutations. Cerebral organoids were exposed to human serum to induce SAD features.	Increased production of Aβ-42; accumulation of hyperphosphorylated tau; synaptic loss, and impaired neural network.

The studies included cover familial AD mutations, mutations related to SAD, as well as other alternative methods used to induce neurodegeneration in these models. The table details the phenotypic characteristics observed in the organoids, such as Aβ accumulation, P-tau presence, and other pathological markers associated with AD. Although recent studies have emphasized the critical role of astrocytes in AD pathogenesis, it is notable that many organoid models fail to address or study astrocyte-related phenotypes. This oversight could limit the understanding of the full spectrum of neurodegenerative processes in AD, as the involvement of astrocytes is increasingly recognized as a significant factor in disease progression.

Furthermore, gene-editing techniques such as CRISPR/Cas9 and viral vectors like AAV-mediated gene transfer, have been employed to introduce tau mutations like tau-P301L to promote tau aggregation in organoids [135]. However, these mutations are commonly associated with other tauopathies rather than AD and may therefore provide insights into general tau aggregation mechanisms instead of AD-specific tau pathology.

Recent advancements using astrocyte-neuron spheroids have shown potential in modeling tau propagation accurately. In these models, P-tau can spread from pre-treated to untreated neurons, providing a closer approximation of tau's self-propagation in a more complex cellular environment [136].

Additional research addresses AD pathology by exploring alternative signaling pathways (Table 1). Using the compound Aftin-5, Pavoni et al. managed to chemically induce an AD model, triggering a notable increase in Aβ production in organoids derived from healthy patients [137]. In another study, Cairns and colleagues investigated the potential role of herpes simplex virus type 1 (HSV-1) in promoting AD-related pathologies in neural spheroids cultured on porous silk scaffolds [138].

This line of inquiry stems from pioneering work by the Itzhaki laboratory, which revealed significantly elevated levels of Herpesviridae DNA in amyloid plaques extracted from postmortem brain tissue of patients with sporadic AD [139]. A recent investigation demonstrated that brain organoids infected with HSV-1 faithfully reproduced the neuropathology associated with the accumulation of Aβ plaques [140]. This included the formation of multicellular Aβ deposits, alteration in the regulation of endogenous mediators related to AD, reactive gliosis, the presence of neuroinflammation and neuronal loss [140]. Furthermore, the inhibition of the mitochondrial enzyme called pitrilysin metallopeptidase 1 (PITRM1) resulted in a significant increase in the aggregation of Aβ, P-tau and the appearance of neuronal apoptosis in cortical organoids derived from iPSCs [99]. Overall, these alternative models provide unique advantages and mechanisms to characterize AD without the genetic modifications associated with FAD or SAD [137,141,142].

### 4.2 Limitations related to epigenome preservation

Recent advancements in AD modeling have brought to light the importance of preserving epigenetic integrity, revealing key limitations of current methodologies. While iPSCs have revolutionized disease modeling by enabling the generation of various human cell types, they present challenges, particularly the loss of age-related epigenetic signatures upon reprogramming somatic cells. This cellular rejuvenation can obscure the age-dependent aspects of neurodegenerative diseases like AD [155-159], limiting the ability of iPSC-derived models to fully capture relevant aging processes and disease phenotypes [160-162].

In light of these limitations, recent research has explored alternative methodologies that preserve epigenetic fidelity. Mertens et al. (2022) elucidated that neurons directly induced from fibroblasts of AD patients retain critical disease-related epigenetic modifications, which are lost when fibroblasts are reprogrammed into iPSCs [163]. Their findings emphasize that these epigenetic features are crucial for understanding AD pathology and are preserved in models bypassing iPSC reprogramming. Following this approach, Sun et al. (2024) introduced direct neuronal reprogramming techniques combined with 3D spheroid cultures, offering a more accurate model of AD neuropathology while conserving disease-relevant epigenetic attributes [164]. The retention of these modifications in 3D models provides a more nuanced understanding of disease progression. Together, these studies emphasize the necessity of preserving epigenetic integrity to accurately model AD mechanisms.

### 4.3 Vasculature deficiency in the context of AD

**Figure 5.**
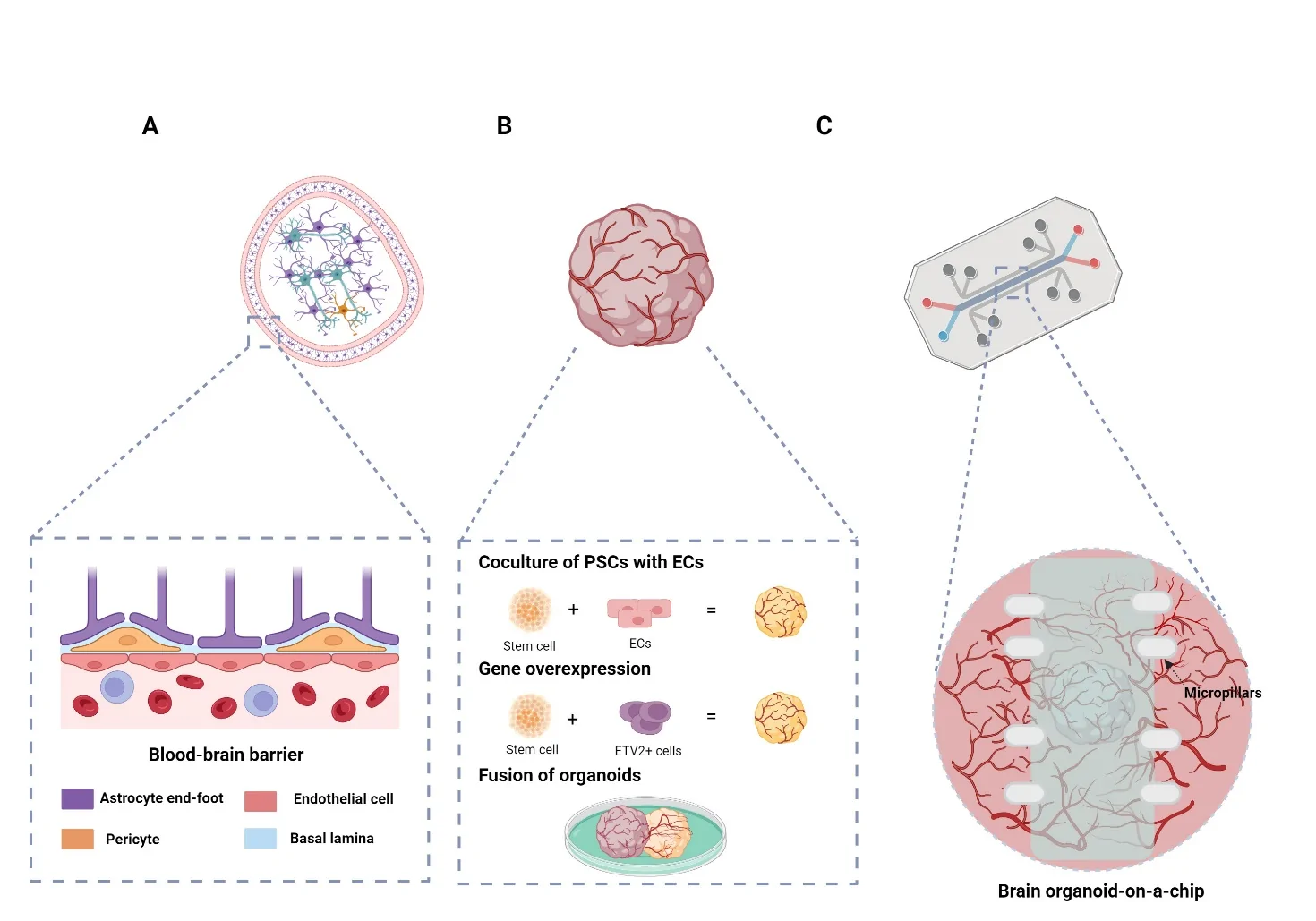
**Strategies to alleviate hypoxia and metabolic stress in brain organoids: Blood-Brain Barrier models, vascularization, and microfluidic integration**. In brain organoids, as mentioned in the text, the central regions often experience limited glucose and oxygen supply due to the dense cellular architecture, leading to a hypoxic environment [165]. This scarcity triggers the activation of the Warburg effect, where cells shift from oxidative phosphorylation to anaerobic glycolysis to produce energy. Although less efficient, this metabolic adaptation allows cells to survive by rapidly consuming glucose and producing lactate, even in the presence of oxygen. However, the accumulation of lactate and the acidic microenvironment can lead to cellular stress, apoptosis, and eventual necrosis in the organoid's core. To address these challenges, the incorporation of BBB models (A), the introduction of blood vessels formed from endothelial cells (ECs) (B) and microfluidic chips (C), offer promising solutions. These approaches enhance nutrient and oxygen distribution, reduce hypoxia, and limit the reliance on the Warburg effect, thereby preserving cellular viability, supporting complex tissue architecture, and mimicking more accurately the in vivo conditions of the human brain. Created with BioRender.com.

Despite the impressive tissue architecture provided by the three-dimensional structure of brain organoids, a significant obstacle arises in the form of nutrient and oxygen deficiency in the organoid's core due to the lack of microvasculature [165]. Certain cells, such as radial glia, can maintain their viability even in the innermost layers of the organoid. This may be due to their unique metabolic requirements and their elongated morphology, which allow them to interact with the external environment. However, as the neuronal population differentiates and increases, these cells are gradually displaced inward and experience necrosis due to the lack of nutrients. Significant advances have been made in improving the survival of brain organoids, including the administration of growth factors such as BDNF, as well as the transplantation of organoids into a rodent host [166,167]. Transplantation has not only demonstrated unprecedented cellular survival due to effective blood circulation, but it has also shown the neuronal connectivity of the organoid with the rodent brain [167]. These successful in vivo transplants suggest the possibility that brain organoids have an intrinsic potential to establish functional connections. However, this is a labor-intensive strategy requiring specialized skills, making it difficult to conduct comprehensive characterization across multiple samples. To address this challenge in a fully in vitro system, a traditional organotypic slice culture technique has recently been employed [168]. This involves air-liquid interface (ALI) cell culture, which leads to substantial improvements in neuronal maturation and survival as it shows a higher number of cortical neuron populations and a decrease in cell death [169]. Additionally, the expression of human ETS variant 2 transcription factor (hETV2) reprograms human dermal fibroblasts into ECs in the absence of growth factors and induces the formation of vascular-like structures in organoids [170]. This model offers a valuable platform that reduces the apoptotic and hypoxic condition of organoid core. Alternatively, iPSC-derived ECs vascularize developing brain organoids when adhered to their surface [171]. In brief, ECs embedded together along with brain organoids in a Matrigel basement membrane matrix generate tubular structures with CD31+ cells with capillary structures weeks later [171]. Vascularized 3D spheroid models also include a microfluidic chip (Fig. 5). From the microchannels of these devices, angiogenic sprouts or blood vessel-like structures are induced towards the spheroid to form a continuous lumen capable of delivering biological substances to the interior of the organoid [172].

The study by Wang et al. (2021) unveiled significant advancements in the culture of brain organoids using microfluidic chips, enhancing several critical aspects of cellular development. In particular, they observed increased cellular proliferation, homogeneous differentiation of more neural progenitors into neurons and glia, and preservation of cellular heterogeneity resembling in vivo conditions. The chips also reduced hypoxia-related stress and apoptosis. Additionally, organoids showed improved synaptic activity and electrophysiological patterns, indicating greater neuronal maturity and functionality [173]. These findings highlight the considerable potential of microfluidic chips to enhance the quality, complexity, and functionality of brain organoids, opening new avenues in biomedical research.

### 4.4 Lack of blood-brain barrier

Despite the advances in vascularizing brain organoids, replicating the intricate structure and function of the BBB remains challenging. Recent developments made feasible the integration of astrocytes and pericytes, which are essential for BBB integrity, into brain organoids. In these advanced models, BBB spheroids exhibit high expression of tight junction proteins on their surface, along with VEGF-dependent permeability, efflux pump activity, and receptor-mediated transcytosis of angiopep-2 [174-176]. Therefore, this setup successfully replicates key aspects of BBB functionality, enabling accurate assessment of drug permeability and efficacy. Additionally, these models are valuable for studying AD, as BBB leakage is a well-known risk factor for the condition [177-181]. By exposing brain organoids to human serum, researchers can mimic the consequences of BBB breakdown observed in AD patients [142,182]. This approach has been shown to recapitulate AD-like pathologies, including increased Aβ aggregates, elevated levels of p-tau, synaptic loss, and impaired neural networks. This exposure also induces elevated levels of beta-secretase 1 (BACE) and glycogen synthase kinase-3 alpha/beta (GSK3α/β), further elucidating the mechanisms underlying AD [142]. In turn, microfluidic devices offer a more dynamic and realistic representation of the BBB by introducing blood flow and simulating the shear stress that occurs at the barrier. These advancements enhance the physiological relevance of BBB models in culture, providing a closer approximation to in vivo conditions. However, a significant limitation of these systems is their requirement for specialized equipment and technical expertise, which hampers their broader application within the neuroscience research community.

### 4.5 Lack of immune system

Upon injury, microglia rapidly extend their processes towards the affected area, migrating to the lesion site to recognize pathogens, ramify, and initiate an immune response [183]. In AD brains and mouse models, microglia are frequently observed in close proximity to amyloid plaques, exhibiting an 'activated' proinflammatory phenotype [184,185]. Initially believed to contribute to amyloid deposition, subsequent electron microscopy studies revealed that microglia can engulf Aβ peptides through endosome-like compartments [186,187]. In vitro experiments utilizing radioisotope or fluorescent-labeled Aβ and direct injection of fibrillar Aβ into rodent brains have further demonstrated microglia's capability to internalize the peptide [188-190]. Integrating microglia into brain organoids enhances the capability to model debris clearance and study inflammatory responses. A recent preprint illustrates how interactions between microglia and astrocytes within a 3D neurosphere effectively reduce Aβ toxicity. Additionally, this study shows that iPSC-derived primitive macrophages, resembling microglia, significantly reduce Aβ aggregation and promote neuronal survival during Aβ treatment in a human 3D neurospheres. These findings suggest that the activation state of microglia-like cells remains consistent across various model systems and underscores the importance of modeling interactions with peripheral immune cells to elicit robust pro-inflammatory responses.

The regional progression of brain atrophy in AD is strongly correlated with tau accumulation [191-193]. In this context, Chen et al. (2023) investigated the role of T cells in neurodegeneration observed in tauopathies. Their study reveals that microglia are essential in recruiting T cells to the brain in response to tau aggregation [194]. Infiltration of T cells exacerbates neurodegeneration and promotes a pro-inflammatory environment. By blocking T cell entry or activation, the researchers observed a significant reduction in neurodegeneration in mouse models of tauopathy. These findings suggest that targeting T cells could offer a novel therapeutic approach for mitigating neurodegenerative diseases like AD, presenting an alternative to traditional treatments focused on tau or microglial dysfunction.

Building on these findings, another study developed a sophisticated 3D human tri-culture system comprising neurons, astrocytes, and microglia derived from iPSCs [195]. This model successfully recapitulates key aspects of AD pathology, including Aβ aggregation, tau hyperphosphorylation, and neuronal death. Within this system, microglia exhibit an activated phenotype, characterized by increased expression of pro-inflammatory cytokines and enhanced phagocytic activity. Interestingly, while iPSCs-derived microglia play a beneficial role by clearing amyloid plaques and other debris, they also contribute to neuroinflammation and neuronal damage through the release of pro-inflammatory cytokines [195]. This dual role highlights the complexity of microglial function in AD and the importance of targeting their activation states to mitigate harmful effects while enhancing protective functions.

Recent studies have also explored the role of TREM2, a key receptor involved in microglial function, within organoids. For example, it has been demonstrated that brain organoids with TREM2-deficient microglia exhibit impaired phagocytic activity and altered immune responses, exacerbating AD pathology [196]. This highlights TREM2’s critical role in regulating microglial function and emphasizes the utility of organoid models in elucidating the molecular mechanisms underlying microglia-mediated contribution to neurodegenerative diseases like AD

### 4.6 Lack of myelination

A decline in myelination and the subsequent impairment of oligodendrocyte-axon signaling are increasingly associated with neurodegenerative disorders, such as amyotrophic lateral sclerosis (ALS), PD and AD [197-199]. Hypomyelination also manifests in various neurodevelopmental conditions like DS and is characteristic of leukodystrophies [200,201]. Current efforts focus on enhancing OL function and survival, including the delivery of exogenous oligodendrocyte progenitor cells (OPCs). These approaches aim to restore axonal integrity and neurological function, with potential applications in AD research. However, significant challenges remain in achieving effective differentiation and maturation of OLs in brain organoids, where conventional methods often fail to support their development [202].

A recent study achieved relative robust induction of oligodendrogenesis and myelination, albeit after a prolonged glial differentiation protocol for 210 days [203]. Such extended differentiation timelines make experimentation difficult and impractical. To address this, alternative methods have been developed to accelerate OL specification in organoids by shortening the protocol to 12 weeks of culture [204]. The addition of PDGF-AA and IGF-1 similarly achieved mature OLs by day 100 [205]. Additionally, innovative and simplified one-step protocols for generating human cortical brain organoids further streamlined myelinating oligodendrocyte generation to within just 42 days. However, the organoids exhibited only a few axons with compacted myelin sheaths [206]. This limited myelination may improve with more prolonged culture times or the addition of myelination-enhancing molecules.

Considering that the APOE4 gene is associated with increased susceptibility to demyelination, the study of myelinated organoids could be particularly promising for investigating these complex dynamics in AD. Recent studies have shown that the presence of APOE4 in astrocytes induces significant disruption in lipid balance, affecting essential cellular functions and increasing the risk of demyelination [207-209]. On the other hand, treatment with docosahexaenoic acid has shown potential in promoting remyelination. DHA can protect and restore damaged myelin in experimental models, suggesting it may be useful in counteracting the demyelinating effects of APOE4 [210]. Thus, the innovation in accelerating OL specification and myelination in organoid models is not only a technical advancement but also a pivotal step towards unraveling the complexities of myelination and developing targeted therapies for demyelinating diseases.

## Future prospects

While brain organoids derived from human iPSCs have markedly enhanced our capacity to model neurodevelopmental processes and investigate neurodegenerative diseases such as AD, it is essential to recognize the inherent limitations of these culture systems. These constraints bear significant implications for the accuracy, reproducibility, and translational relevance of the insights gleaned from such models.

One of the most notable limitations is the insufficient functional maturity exhibited by brain organoids. These organoids can faithfully recapitulate critical aspects of early human neurodevelopment, encompassing the genesis, proliferation, and differentiation of neural progenitors into neurons and glial cells. However, they frequently fall short of replicating the complex, multilayered architecture and the full spectrum of functional characteristics inherent to adult human brain tissue [105, 108, 211-213]. This developmental immaturity poses significant challenges for modeling late-onset neurodegenerative diseases, where age-related processes are central to disease pathogenesis. Considering that aging is the primary risk factor for AD, the extent to which brain organoids can be utilized to model age-related neurodegeneration remains uncertain. This underscores the need for rigorous validation of disease-relevant phenotypes. To address these limitations and enhance their relevance for AD research, strategies such as integrating brain organoids with models that simulate neuroinflammatory conditions or BBB dysfunction, as mentioned earlier, may be useful. Additionally, the use of chronic exposure to amyloidogenic peptides, neurotoxins, or stressors that mimic aging processes within organoids may help to induce features more representative of the disease’s late-onset nature. These strategies could provide a more accurate platform for investigating AD pathology and testing potential therapeutic interventions. However, as indicated, thorough validation will be necessary to confirm that the disease phenotypes are faithfully reproduced.

In addition to the challenges associated with functional maturity, another critical aspect to consider with brain organoids is their intrinsic heterogeneity. The generation of organoids often results in variability in size, cellular composition, and structural organization [102]. This heterogeneity can lead to inconsistencies in experimental outcomes, thereby complicating the reproducibility and interpretation of results, particularly in studies aimed at modeling complex diseases like AD. It is therefore essential to develop refined protocols promoting uniform organoid formation. Reducing variability through the selection of guided differentiation methods could further enhance the consistency and reliability of organoid-based models. Moreover, as previously mentioned, the use of complex ECMs substitutes, like Matrigel, significantly contributes to this variability. Although Matrigel provides a versatile and affordable scaffold for organoid culture, its inherent complexity with over 1800 unique proteins, introduces a level of unpredictability [214]. The undefined nature of Matrigel complicates the identification of specific signals necessary for consistent organoid structure and function, and this challenge is further exacerbated by lot-to-lot variations. Such inconsistencies underscore the importance of exploring more defined or synthetic alternatives that could offer a more controlled microenvironment. In addition, organoids remain intrinsically reductionist models as they partially replicate key aspects of brain physiology, such as myelination, microglial activity, and vascularization. The absence of these crucial elements constrains the organoids’ capacity to emulate fully the complex in vivo environment of the human brain. As discussed in this review, current research is addressing these gaps by integrating microglial cells and vascular networks through advanced bioengineering techniques, as well as protocols that promote OL maturation. The incorporation of microglia, in particular, is anticipated to facilitate the regeneration of myelin since recent findings demonstrate that its absence can lead to CNS demyelination with increasing age [215]. This highlights the potential for this cell type integration to support myelin repair and improve the accuracy of organoid models in studying AD. Similarly, the incorporation of a functional vascular network into brain organoids, though presenting considerable challenges, is essential for enhancing their physiological fidelity. The absence of a functional vasculature not only restricts nutrient and oxygen diffusion but also omits critical neurovascular interactions that influence neuronal function, glial support, and BBB integrity. Thus, methods such as spinning bioreactors, the induction of ECs and perivascular cells, and the integration of models that replicate the BBB, including microfluidic chips, are being explored.

The fusion of organoids to create "assembloids" offers a promising approach to overcome the limitations of traditional, reductionist brain models. In particular, a cortical-blood vessel assembloids that successfully replicated AD phenotypes was developed [216]. By integrating cortical organoids with vascular elements, the model allowed for the study of critical neuron-glia-vascular interactions. Following SARS-CoV-2 infection, these assembloids exhibited glial activation and other AD-like features, demonstrating their utility in exploring complex disease mechanisms and potential therapeutic targets. Moreover, assembloids can also be engineered to replicate the intricate neuronal connections implicated in AD that alter large-scale brain networks, particulary those involved in memory formation [217,218]. These changes encompass a deceleration of the alpha rhythm toward theta frequencies, aberrant gamma activity, and a reduction in slow-wave oscillations during sleep [219-221]. It would be particularly compelling to leverage advanced tools such as multi-electrode arrays within assembloid models to investigate these specific electrophysiological disruptions. This approach could offer significant insights into how hyperactivity in local networks, along with impaired connectivity between key brain regions such as the hippocampus and the cortex, contributes to the cognitive dysfunction observed in AD pathology [136]. Furthermore, assembloid models provide a promising platform for studying the propagation of tau protein across neuronal circuits, particularly its spread from the entorhinal cortex to the hippocampus and other neocortical areas [222-224]. Understanding these mechanisms is crucial for unraveling the progression of tau pathology and its role in driving the disease. Following this line of thought and considering the emerging hypothesis that gut-derived infective agents may influence AD progression, incorporating gut-brain assembloids could be highly informative. These gut-brain assembloids could facilitate the investigation of how pathogenic microbes or inflammatory signals originating in the gut might contribute to AD pathology. Such models could provide new insights into the gut-brain axis and its role in neurodegeneration, particularly in understanding how gut-derived infections or dysbiosis could trigger or exacerbate amyloid plaque formation and tau pathology in the brain.

From a practical perspective, brain organoids require significant time to develop and mature, which can increase costs and reduce experimental efficiency. Therefore, exploring methods to accelerate this maturation process is crucial. For example, NOTCH inhibitors effectively speed up neuronal differentiation in vitro and conceivably in brain organoids [225]. Additionally, ensuring that all cells within the organoid reach a similar level of maturity is vital for accurate disease modeling. Techniques such as single-cell RNA sequencing may help verify this uniformity. Specifically, it is important to monitor gene expression in astrocytes in AD organoids as these cells are the primary cells expressing APOE in the adult brain. Proper expression of these genes is crucial for studying AD and ensuring that organoids accurately model late-onset pathology.

In conclusion, while brain organoids have advanced our understanding of AD, significant challenges remain. Nevertheless, with ongoing improvements in functional maturity, uniformity, and the incorporation of key physiological elements, we anticipate that these models will become increasingly robust. Overcoming these challenges will enable the development of more accurate platforms for investigating AD and testing therapeutic interventions, ultimately bringing us closer to effective treatments.

## References

[1] Sasai Y, Eiraku M, Suga H (2012). In vitro organogenesis in three dimensions: self-organising stem cells. Development, 139(22):4111-21. 10.1242/dev.079590 23093423

[2] Lancaster MA, Knoblich JA (2014). Organogenesis in a dish: modeling development and disease using organoid technologies. Science, 18;345(6194):1247125 10.1126/science.1247125 25035496

[3] Clevers H (2016). Modeling Development and Disease with Organoids. Cell, 165(7):1586-97. 10.1016/j.cell.2016.05.082 27315476

[4] Kim J, Koo BK, Knoblich JA (2020). Human organoids: model systems for human biology and medicine. Nat Rev Mol Cell Biol, 21(10):571-84. 10.1038/s41580-020-0259-3 PMC7339799 32636524

[5] Fujii M, Sato T (2021). Somatic cell-derived organoids as prototypes of human epithelial tissues and diseases. Nat Mater, 20(2):156-69. 10.1038/s41563-020-0754-0 32807924

[6] De Strooper B, Karran E (2016). The Cellular Phase of Alzheimer’s Disease. Cell, 164(4):603-15. 10.1016/j.cell.2015.12.056 26871627

[7] Ginsberg SD, Che S, Counts SE, Mufson EJ (2006). Single cell gene expression profiling in Alzheimer’s disease. NeuroRx, 3(3):302-18. 10.1016/j.nurx.2006.05.007 PMC3593387 16815214

[8] Mattsson N, Schott JM, Hardy J, Turner MR, Zetterberg H (2016). Selective vulnerability in neurodegeneration: insights from clinical variants of Alzheimer’s disease. J Neurol Neurosurg Psychiatry, 87(9):1000-4. 10.1136/jnnp-2015-311321 26746185

[9] Yue C, Jing N (2005). The promise of stem cells in the therapy of Alzheimer’s disease. Transl Neurodegener, 28;4:8. 10.1186/s40035-015-0029-x PMC4423588 25954503

[10] Glenner GG, Wong CW (1984). Alzheimer’s disease and Down’s syndrome: Sharing of a unique cerebrovascular amyloid fibril protein. Biochem Biophys Res Commun, 16;122(3):1131-5. 10.1016/0006-291x(84)91209-9 6236805

[11] Glenner GG WCQVEED (1984). The amyloid deposits in Alzheimer’s disease: their nature and pathogenesis. App Pathol, 2(6):357-69. 6242724

[12] Masters CL, Simms G, Weinman NA, Multhaup G, McDonald BL, Beyreuther K (1985). Amyloid plaque core protein in Alzheimer disease and Down syndrome. Proc Natl Acad Sci U S A, 82(12):4245-9. 10.1073/pnas.82.12.4245 PMC397973 3159021

[13] Lai F, Williams RS (1989). A prospective study of Alzheimer disease in Down syndrome. Arch Neurol. 46(8):849-53. 10.1001/archneur.1989.00520440031017 2527024

[14] Wisniewski KE, Wisniewski HM, Wen GY (1985). Occurrence of neuropathological changes and dementia of Alzheimer’s disease in Down’s syndrome. Ann Neurol, 17(3):278-82. 10.1002/ana.410170310 3158266

[15] Goldgaber D, Lerman MI, McBride OW, Saffiotti U, Gajdusek DC (1987). Characterization and chromosomal localization of a cDNA encoding brain amyloid of Alzheimer’s disease. Science, 235(4791):877-80. 10.1126/science.3810169 3810169

[16] Kang J, Lemaire HG, Unterbeck A, Salbaum JM, Masters CL, Grzeschik KH, et al. (1987). The precursor of Alzheimer’s disease amyloid A4 protein resembles a cell-surface receptor. Nature, 325(6106):733-6. 10.1038/325733a0 2881207

[17] Hardy JA, Higgins GA (1992). Alzheimer’s disease: the amyloid cascade hypothesis. Science, 256(5054):184-5. 10.1126/science.1566067 1566067

[18] Hardy J, Hardy CJ, Lila R (2017). The discovery of Alzheimer-causing mutations in the APP gene and the formulation of the “amyloid cascade hypothesis”. FEBS J, 284(7):1040-4. 10.1111/febs.14004 28054745

[19] Selkoe DJ (2011). Resolving controversies on the path to Alzheimer’s therapeutics. Nat Med, 17(9):1060-5. 10.1038/nm.2460 21900936

[20] Hanseeuw BJ, Betensky RA, Jacobs HIL, Schultz AP, Sepulcre J, Becker JA, et al. (2019). Association of Amyloid and Tau with Cognition in Preclinical Alzheimer Disease: A Longitudinal Study. JAMA Neurol, 76(8):915-24. 10.1001/jamaneurol.2019.1424 PMC6547132 31157827

[21] Sperling RA, Mormino EC, Schultz AP, Betensky RA, Papp K V., Amariglio RE, et al (2019). The impact of amyloid-beta and tau on prospective cognitive decline in older individuals. Ann Neurol, 85(2):181-93. 10.1002/ana.25395 PMC6402593 30549303

[22] Ullian EM, Sapperstein SK, Christopherson KS, Barres BA (2021). Control of synapse number by glia. Science, 291(5504):657-61. 10.1126/science.291.5504.657 11158678

[23] Pannasch U, Rouach N (2013). Emerging role for astroglial networks in information processing: from synapse to behavior. Trends Neurosci, 36(7):405-17. 10.1016/j.tins.2013.04.004 23659852

[24] Sofroniew M V., Vinters H V (2010). Astrocytes: Biology and pathology. Acta Neuropathol, 119(1):7-35. 10.1007/s00401-009-0619-8 PMC2799634 20012068

[25] Scuderi C, Stecca C, Iacomino A, Steardo L (2013). Role of astrocytes in major neurological disorders: the evidence and implications. IUBMB Life, 65(12):957-61. 10.1002/iub.1223 24376207

[26] Rossi D (2015). Astrocyte physiopathology: At the crossroads of intercellular networking, inflammation and cell death. Prog Neurobiol, 130:86-120. 10.1016/j.pneurobio.2015.04.003 25930681

[27] Armstrong RA (2009). The molecular biology of senile plaques and neurofibrillary tangles in Alzheimer’s disease. Folia Neuropathology, 47(4):289-99. 20054780

[28] Rodríguez JJ, Olabarria M, Chvatal A, Verkhratsky A (2009). Astroglia in dementia and Alzheimer’s disease. Cell Death Differ, 16(3):378-85. 10.1038/cdd.2008.172 19057621

[29] Li S, Jin M, Koeglsperger T, Shepardson NE, Shankar GM, Selkoe DJ (2011). Soluble Aβ Oligomers Inhibit Long-Term Potentiation through a Mechanism Involving Excessive Activation of Extrasynaptic NR2B-Containing NMDA Receptors. The Journal of Neuroscience, 31(18):6627. 10.1523/JNEUROSCI.0203-11.2011 PMC3100898 21543591

[30] Jones VC, Atkinson-Dell R, Verkhratsky A, Mohamet L (2019). Aberrant iPSC-derived human astrocytes in Alzheimer's disease. Cell Death Dis, 10(3):244. 10.1038/s41419-019-1422-7 PMC6414541 30862780

[31] Fünfschilling U, Supplie LM, Mahad D, Boretius S, Saab AS, Edgar J, et al. (2012). Glycolytic oligodendrocytes maintain myelin and long-term axonal integrity. Nature, 485(7399):517-21. 10.1038/nature11007 PMC3613737 22622581

[32] Lee S, Viqar F, Zimmerman ME, Narkhede A, Tosto G, Benzinger TLS, et al. (2016). White matter hyperintensities are a core feature of Alzheimer’s disease: Evidence from the Dominantly Inherited Alzheimer Network. Ann Neurol, 79(6):929. 10.1002/ana.24647 PMC4884146 27016429

[33] Jansen IE, Savage JE, Watanabe K, Bryois J, Williams DM, Steinberg S, et al. (2019). Genome-wide meta-analysis identifies new loci and functional pathways influencing Alzheimer’s disease risk. Nat Genet, 51(3):404-13. 10.1038/s41588-018-0311-9 PMC6836675 30617256

[34] Jiang T, Yu JT, Zhu XC, Tan L (2013). TREM2 in Alzheimer’s disease. Mol Neurobiol, 48(1):180-5. 10.1007/s12035-013-8424-8 23407992

[35] Zhong L, Chen XF (2019). The Emerging Roles and Therapeutic Potential of Soluble TREM2 in Alzheimer’s Disease. Front Aging Neurosci, 26;11:328. 10.3389/fnagi.2019.00328 PMC6988790 32038221

[36] Hickman SE, El Khoury J (2014). TREM2 and the neuroimmunology of Alzheimer’s disease. Biochem Pharmacol, 88(4):495. 10.1016/j.bcp.2013.11.021 PMC3972304 24355566

[37] Qin Q, Teng Z, Liu C, Li Q, Yin Y, Tang Y (2021). TREM2, microglia, and Alzheimer’s disease. Mech Ageing Dev, 195:111438. 10.1016/j.mad.2021.111438 33516818

[38] Wood H (2020). TREM2 activation promotes remyelination. Nat Rev Neurol, 16(10):522. 10.1038/s41582-020-0404-9 32839583

[39] Cantoni C, Bollman B, Licastro D, Xie M, Mikesell R, Schmidt R, et al. (2015). TREM2 regulates microglial cell activation in response to demyelination in vivo. Acta Neuropathol, 129(3):429-47. 10.1007/s00401-015-1388-1 PMC4667728 25631124

[40] Zhang Y, Sloan SA, Clarke LE, Caneda C, Plaza CA, Blumenthal PD, et al. (2016). Purification and characterization of progenitor and mature human astrocytes reveals transcriptional and functional differences with mouse. Neuron, 89(1):37. 10.1016/j.neuron.2015.11.013 PMC4707064 26687838

[41] Srinivasan K, Friedman BA, Larson JL, Lauffer BE, Goldstein LD, Appling LL, et al. (2016). Untangling the brain’s neuroinflammatory and neurodegenerative transcriptional responses. Nat Commun, 21;7:11295. 10.1038/ncomms11295 PMC4844685 27097852

[42] Obermeier B, Daneman R, Ransohoff RM (2013). Development, maintenance and disruption of the blood-brain barrier. Nat Med, 19(12):1584-96. 10.1038/nm.3407 PMC4080800 24309662

[43] Daneman R, Zhou L, Kebede AA, Barres BA (2010). Pericytes are required for blood-brain barrier integrity during embryogenesis. Nature, 468(7323):562-6. 10.1038/nature09513 PMC3241506 20944625

[44] Vanlandewijck M, He L, Mäe MA, Andrae J, Ando K, Del Gaudio F, et al. (2018). A molecular atlas of cell types and zonation in the brain vasculature. Nature, 554(7693):475-80. 10.1038/nature25739 29443965

[45] Chow BW, Gu C (2015). The Molecular Constituents of the Blood-Brain Barrier. Trends Neurosci, 38(10):598-608. 10.1016/j.tins.2015.08.003 PMC4597316 26442694

[46] Profaci CP, Munji RN, Pulido RS, Daneman R (2020). The blood-brain barrier in health and disease: Important unanswered questions. J Exp Med, 217(4):e20190062. 10.1084/jem.20190062 PMC7144528 32211826

[47] Kisler K, Nelson AR, Montagne A, Zlokovic B V (2017). Cerebral blood flow regulation and neurovascular dysfunction in Alzheimer disease. Nature Reviews, 18(7):419-34. 10.1038/nrn.2017.48 PMC5759779 28515434

[48] Prineas JW, Parratt JDE, Kirwan PD (2016). Fibrosis of the Choroid Plexus Filtration Membrane. J Neuropathol Exp Neurol, 75(9):855-67. 10.1093/jnen/nlw061 PMC5015658 27444353

[49] Kaur C, Rathnasamy G, Ling EA (2016). The Choroid Plexus in Healthy and Diseased Brain. J Neuropathol Exp Neurol, 75(3):198-213. 10.1093/jnen/nlv030 26888305

[50] Brkic M, Balusu S, Van Wonterghem E, Gorlé N, Benilova I, Kremer A, et al. (2015). Amyloid β Oligomers Disrupt Blood-CSF Barrier Integrity by Activating Matrix Metalloproteinases. J Neurosci, 35(37):12766-78. 10.1523/JNEUROSCI.0006-15.2015 PMC6795210 26377465

[51] Masseguin C, LePanse S, Corman B, Verbavatz JM, Gabrion J (2005). Aging affects choroidal proteins involved in CSF production in Sprague-Dawley rats. Neurobiol Aging, 26(6):917-27. 10.1016/j.neurobiolaging.2004.07.013 15718051

[52] Kant S, Stopa EG, Johanson CE, Baird A, Silverberg GD (2018). Choroid plexus genes for CSF production and brain homeostasis are altered in Alzheimer’s disease. Fluids Barriers CNS, 15(1):34. 10.1186/s12987-018-0120-7 PMC6291926 30541599

[53] Bakken TE, Jorstad NL, Hu Q, Lake BB, Tian W, Kalmbach BE, et al. (2021). Comparative cellular analysis of motor cortex in human, marmoset and mouse. Nature, 98(7879):111-9. 10.1038/s41586-021-03465-8 PMC8494640 34616062

[54] Castillo E, Leon J, Mazzei G, Abolhassani N, Haruyama N, Saito T, et al. (2017). Comparative profiling of cortical gene expression in Alzheimer’s disease patients and mouse models demonstrates a link between amyloidosis and neuroinflammation. Scientific Reports, 7(1):1-16. 10.1038/s41598-017-17999-3 PMC5736730 29259249

[55] Geirsdottir L, David E, Keren-Shaul H, Weiner A, Bohlen SC, Neuber J, et al. (2019). Cross-Species Single-Cell Analysis Reveals Divergence of the Primate Microglia Program. Cell, 179(7):1609-1622.e16. 10.1016/j.cell.2019.11.010 31835035

[56] Song HW, Foreman KL, Gastfriend BD, Kuo JS, Palecek SP, Shusta E V (2020). Transcriptomic comparison of human and mouse brain microvessels. Scientific Reports, 10(1):1-14. 10.1038/s41598-020-69096-7 PMC7378255 32704093

[57] Yang AC, Vest RT, Kern F, Lee DP, Agam M, Maat CA, et al. (2022). A human brain vascular atlas reveals diverse mediators of Alzheimer’s risk. Nature, 603(7903):885-92. 10.1038/s41586-021-04369-3 PMC9635042 35165441

[58] Oberheim NA, Wang X, Goldman S, Nedergaard M (2006). Astrocytic complexity distinguishes the human brain. Trends Neurosci, 29(10):547-53. 10.1016/j.tins.2006.08.004 16938356

[59] Oberheim NA, Takano T, Han X, He W, Lin JHC, Wang F, et al. (2009). Uniquely hominid features of adult human astrocytes. J Neurosci, 29(10):3276-87. 10.1523/JNEUROSCI.4707-08.2009 PMC2819812 19279265

[60] Li J, Pan L, Pembroke WG, Rexach JE, Godoy MI, Condro MC, et al. (2021). Conservation and divergence of vulnerability and responses to stressors between human and mouse astrocytes. Nat Commun, 12(1):1-20. 10.1038/s41467-021-24232-3 PMC8233314 34172753

[61] Jahn O, Siems SB, Kusch K, Hesse D, Jung RB, Liepold T, et al. (2020). The CNS Myelin Proteome: Deep Profile and Persistence After Post-mortem Delay. Front Cell Neurosci, 19;14:239. 10.3389/fncel.2020.00239 PMC7466725 32973451

[62] Gargareta VI, Reuschenbach J, Siems SB, Sun T, Piepkorn L, Mangana C, et al. (2022). Conservation and divergence of myelin proteome and oligodendrocyte transcriptome profiles between humans and mice. Elife, 11;11:e77019. 10.7554/eLife.77019 PMC9094742 35543322

[63] Braidy N, Muñoz P, Palacios AG, Castellano-Gonzalez G, Inestrosa NC, Chung RS, et al. (2012). Recent rodent models for Alzheimer’s disease: clinical implications and basic research. J Neural Transm, 119(2):173-95. 10.1007/s00702-011-0731-5 22086139

[64] Uno H, Walker LC (1993). The age of biosenescence and the incidence of cerebral beta-amyloidosis in aged captive rhesus monkeys. Ann N Y Acad Sci, 695(1):232-5. 10.1111/j.1749-6632.1993.tb23058.x 8239288

[65] Voytko M Lou (1999). Impairments in acquisition and reversals of two-choice discriminations by aged rhesus monkeys. Neurobiol Aging, 20(6):617-27. 10.1016/s0197-4580(99)00097-4 10674427

[66] Nakamura S, Nakayama H, Goto N, One F, Sakakibara I, Yoshikawa Y (1998). Histopathological studies of senile plaques and cerebral amyloidosis in cynomolgus monkeys. J Med Primatol, 27(5):244-52. 10.1111/j.1600-0684.1998.tb00244.x 9926980

[67] Buckwalter MS, Wyss-Coray T (2004). Modelling neuroinflammatory phenotypes in vivo. J Neuroinflamm, 1;1(1):10. 10.1186/1742-2094-1-10 PMC500895 15285805

[68] Forny-Germano L, Lyra E Silva NM, Batista AF, Brito-Moreira J, Gralle M, Boehnke SE, et al. (2014). Alzheimer’s disease-like pathology induced by amyloid-β oligomers in nonhuman primates. J Neurosci, 34(41):13629-43. 10.1523/JNEUROSCI.1353-14.2014 PMC6608380 25297091

[69] Li W, Wu Y, Min F, Li Z, Huang J, Huang R (2010). A nonhuman primate model of Alzheimer’s disease generated by intracranial injection of amyloid-β42 and thiorphan. Metab Brain Dis, 25(3):277-84. 10.1007/s11011-010-9207-9 20838863

[70] Gary C, Lam S, Hérard AS, Koch JE, Petit F, Gipchtein P, et al. (2019). Encephalopathy induced by Alzheimer brain inoculation in a non-human primate. Acta Neuropathol Commun, 7(1):126. 10.1186/s40478-019-0771-x PMC6724379 31481130

[71] Azevedo FAC, Carvalho LRB, Grinberg LT, Farfel JM, Ferretti REL, Leite REP, et al. (2009). Equal numbers of neuronal and nonneuronal cells make the human brain an isometrically scaled-up primate brain. J Comp Neurol, 513(5):532-41. 10.1002/cne.21974 19226510

[72] Erö C, Gewaltig MO, Keller D, Markram H (2018). A Cell Atlas for the Mouse Brain. Front Neuroinform, 19;13:7. 10.3389/fninf.2019.00007 PMC6390506 30837861

[73] Herculano-Houzel S (2011). Brains matter, bodies maybe not: the case for examining neuron numbers irrespective of body size. Ann N Y Acad Sci, 1225(1):191-9. 10.1111/j.1749-6632.2011.05976.x 21535005

[74] Yang S, Hu H, Kung H, Zou R, Dai Y, Hu Y, et al. (2023). Organoids: The current status and biomedical applications. Med Comm, 17;4(3):e274. 10.1002/mco2.274 PMC10192887 37215622

[75] Hu W, Lazar MA (2022). Modelling metabolic diseases and drug response using stem cells and organoids. Nat Rev Endocrinol, 18(12):744-59. 10.1038/s41574-022-00733-z PMC9449917 36071283

[76] Sreenivasamurthy S, Laul M, Zhao N, Kim T, Zhu D (2023). Current progress of cerebral organoids for modeling Alzheimer’s disease origins and mechanisms. Bioeng Transl Med, 8(2):e10378. 10.1002/btm2.10378 PMC10013781 36925717

[77] Van den Heuvel MP, Ardesch DJ, Scholtens LH, de Lange SC, van Haren NEM, Sommer IEC, et al. (2024). Human and chimpanzee shared and divergent neurobiological systems for general and specific cognitive brain functions. Proc Natl Acad Sci U S A, 120(22):e2218565120. 10.1073/pnas.2218565120 PMC10235977 37216540

[78] Eiraku M, Watanabe K, Matsuo-Takasaki M, Kawada M, Yonemura S, Matsumura M, et al. (2008). Self-organized formation of polarized cortical tissues from ESCs and its active manipulation by extrinsic signals. Cell Stem Cell, 3(5):519-32. 10.1016/j.stem.2008.09.002 18983967

[79] Lancaster MA, Renner M, Martin CA, Wenzel D, Bicknell LS, Hurles ME, et al. (2013). Cerebral organoids model human brain development and microcephaly. Nature, 501(7467):373-9. 10.1038/nature12517 PMC3817409 23995685

[80] Camp JG, Badsha F, Florio M, Kanton S, Gerber T, Wilsch-Bräuninger M, et al. (2015). Human cerebral organoids recapitulate gene expression programs of fetal neocortex development. Proc Natl Acad Sci U S A, 112(51):15672-7. 10.1073/pnas.1520760112 PMC4697386 26644564

[81] Wang Y, Jeon H (2022). 3D cell cultures toward quantitative high-throughput drug screening. Trends Pharmacol Sci, 43(7):569-81. 10.1016/j.tips.2022.03.014 35504760

[82] Schutgens F, Clevers H (2020). Human organoids: tools for understanding biology and treating diseases. Annu Rev Pathol, 15:211-34. 10.1146/annurev-pathmechdis-012419-032611 31550983

[83] Rowe RG, Daley GQ (2019). Induced pluripotent stem cells in disease modelling and drug discovery. Nat Rev Genet, 20(7):377-88. 10.1038/s41576-019-0100-z PMC6584039 30737492

[84] Takahashi K, Yamanaka S (2006). Induction of pluripotent stem cells from mouse embryonic and adult fibroblast cultures by defined factors. Cell, 126(4):663-76. 10.1016/j.cell.2006.07.024 16904174

[85] Nakagawa M, Koyanagi M, Tanabe K, Takahashi K, Ichisaka T, Aoi T, et al. (2007). Generation of induced pluripotent stem cells without Myc from mouse and human fibroblasts. Nat Biotechnol, 26(1):101-6. 10.1038/nbt1374 18059259

[86] Takahashi K, Tanabe K, Ohnuki M, Narita M, Ichisaka T, Tomoda K, et al. (2007). Induction of pluripotent stem cells from adult human fibroblasts by defined factors. Cell, 131(5):861-72. 10.1016/j.cell.2007.11.019 18035408

[87] Yoshida GJ (2018). Emerging roles of Myc in stem cell biology and novel tumor therapies. J Exp Clin Cancer Res, 37(1):173. 10.1186/s13046-018-0835-y PMC6062976 30053872

[88] Geoghegan E, Byrnes L (2008). Mouse induced pluripotent stem cells. Int J Dev Biol, 52(8):1015-22. 10.1387/ijdb.082640eg 18956334

[89] Tiscornia G, Vivas EL, Belmonte JCI (2011). Diseases in a dish: modeling human genetic disorders using induced pluripotent cells. Nat Med, 17(12):1570-6. 10.1038/nm.2504 22146428

[90] Kim K, Doi A, Wen B, Ng K, Zhao R, Cahan P, et al. (2010). Epigenetic memory in induced pluripotent stem cells. Nature, 467(7313):285-90. 10.1038/nature09342 PMC3150836 20644535

[91] Shi Y, Inoue H, Wu JC, Yamanaka S (2017). Induced pluripotent stem cell technology: a decade of progress. Nat Rev Drug Discov, 16(2):115-30. 10.1038/nrd.2016.245 PMC6416143 27980341

[92] Yagi T, Ito D, Okada Y, Akamatsu W, Nihei Y, Yoshizaki T, et al. (2011) Modeling familial Alzheimer’s disease with induced pluripotent stem cells. Hum Mol, 20(23):4530-9. 10.1093/hmg/ddr394 21900357

[93] Koch P, Tamboli IY, Mertens J, Wunderlich P, Ladewig J, Stüber K, et al. (2012). Presenilin-1 L166P mutant human pluripotent stem cell-derived neurons exhibit partial loss of γ-secretase activity in endogenous amyloid-β generation. Am J Pathol, 180(6):2404-16. 10.1016/j.ajpath.2012.02.012 22510327

[94] Mertens J, Stüber K, Wunderlich P, Ladewig J, Kesavan JC, Vandenberghe R, et al. (2013). APP processing in human pluripotent stem cell-derived neurons is resistant to NSAID-based γ-secretase modulation. Stem Cell Rep, 1(6):491-8. 10.1016/j.stemcr.2013.10.011 PMC3871388 24371804

[95] Woodruff G, Young JE, Martinez FJ, Buen F, Gore A, Kinaga J, et al. (2013). The presenilin-1 ΔE9 mutation results in reduced γ-secretase activity, but not total loss of PS1 function, in isogenic human stem cells. Cell Rep, 5(4):974-85. 10.1016/j.celrep.2013.10.018 PMC3867011 24239350

[96] Sproul AA, Jacob S, Pre D, Kim SH, Nestor MW, Navarro-Sobrino M, et al. (2014). Characterization and Molecular Profiling of PSEN1 Familial Alzheimer’s Disease iPSC-Derived Neural Progenitors. PLoS One, 9(1):e84547. 10.1371/journal.pone.0084547 PMC3885572 24416243

[97] Israel MA, Yuan SH, Bardy C, Reyna SM, Mu Y, Herrera C, et al. (2012). Probing sporadic and familial Alzheimer’s disease using induced pluripotent stem cells. Nature, 482(7384):216-20. 10.1038/nature10821 PMC3338985 22278060

[98] Ochalek A, Mihalik B, Avci HX, Chandrasekaran A, Téglási A, Bock I, et al. (2017). Neurons derived from sporadic Alzheimer’s disease iPSCs reveal elevated TAU hyperphosphorylation, increased amyloid levels, and GSK3B activation. Alzheimers Res Ther, 9(1):1-19. 10.1186/s13195-017-0317-z PMC5709977 29191219

[99] Pérez MJ, Ivanyuk D, Panagiotakopoulou V, Di Napoli G, Kalb S, Brunetti D, et al. (2020). Loss of function of the mitochondrial peptidase PITRM1 induces proteotoxic stress and Alzheimer’s disease-like pathology in human cerebral organoids. Mol Psychiatry, 26(10):5733-50. 10.1038/s41380-020-0807-4 PMC8758476 32632204

[100] Anton D, Burckel H, Josset E, Noel G (2015). Three-Dimensional Cell Culture: A Breakthrough in Vivo. Int J Mol Sci, 16(3):5517-27. 10.3390/ijms16035517 PMC4394490 25768338

[101] Centeno EGZ, Cimarosti H, Bithell A (2018). 2D versus 3D human induced pluripotent stem cell-derived cultures for neurodegenerative disease modelling. Mol Neurodegener, 13(1):1-15. 10.1186/s13024-018-0258-4 PMC5964712 29788997

[102] Qian X, Song H, Ming GL (2019). Brain organoids: advances, applications and challenges. Development, 146(8):dev166074. 10.1242/dev.166074 PMC6503989 30992274

[103] Sasai Y (2013). Next-generation regenerative medicine: organogenesis from stem cells in 3D culture. Cell Stem Cell, 12(5):520-30. 10.1016/j.stem.2013.04.009 23642363

[104] Kadoshima T, Sakaguchi H, Nakano T, Soen M, Ando S, Eiraku M, et al. (2013). Self-organization of axial polarity, inside-out layer pattern, and species-specific progenitor dynamics in human ES cell-derived neocortex. Proc Natl Acad Sci U S A, 110(50):20284-9. 10.1073/pnas.1315710110 PMC3864329 24277810

[105] Lancaster MA, Corsini NS, Wolfinger S, Gustafson EH, Phillips AW, Burkard TR, et al. (2017). Guided self-organization and cortical plate formation in human brain organoids. Nat Biotechnol, 35(7):659-66. 10.1038/nbt.3906 PMC5824977 28562594

[106] Pasca AM, Sloan SA, Clarke LE, Tian Y, Makinson CD, Huber N, et al. (2015). Functional cortical neurons and astrocytes from human pluripotent stem cells in 3D culture. Nat Methods, 12(7):671-8. 10.1038/nmeth.3415 PMC4489980 26005811

[107] Watanabe K, Kamiya D, Nishiyama A, Katayama T, Nozaki S, Kawasaki H, et al. (2005). Directed differentiation of telencephalic precursors from embryonic stem cells. Nat Neurosci, 8(3):288-96. 10.1038/nn1402 15696161

[108] Jo J, Xiao Y, Sun AX, Cukuroglu E, Tran HD, Göke J, et al. (2016). Midbrain-like Organoids from Human Pluripotent Stem Cells Contain Functional Dopaminergic and Neuromelanin-Producing Neurons. Cell Stem Cell, 19(2):248-57. 10.1016/j.stem.2016.07.005 PMC5510242 27476966

[109] Sakaguchi H, Kadoshima T, Soen M, Narii N, Ishida Y, Ohgushi M, et al. (2015). Generation of functional hippocampal neurons from self-organizing human embryonic stem cell-derived dorsomedial telencephalic tissue. Nat Commun, 6(1):1-11. 10.1038/ncomms9896 PMC4660208 26573335

[110] Yoon SJ, Elahi LS, Pașca AM, Marton RM, Gordon A, Revah O, et al. (2019). Reliability of human 3D cortical organoid generation. Nat Methods, 16(1):75. 10.1038/s41592-018-0255-0 PMC6677388 30573846

[111] Kleinman HK, Martin GR (2005). Matrigel: basement membrane matrix with biological activity. Semin Cancer Biol, 15(5):378-86. 10.1016/j.semcancer.2005.05.004 15975825

[112] Huch M, Knoblich JA, Lutolf MP, Martinez-Arias A (2017). The hope and the hype of organoid research. Development, 144(6):938-41. 10.1242/dev.150201 28292837

[113] Soofi SS, Last JA, Liliensiek SJ, Nealey PF, Murphy CJ (2009). The elastic modulus of Matrigel as determined by atomic force microscopy. J Struct Biol, 167(3):216-9. 10.1016/j.jsb.2009.05.005 PMC2747304 19481153

[114] Schneeberger K, Spee B, Costa P, Sachs N, Clevers H, Malda J (2017). Converging biofabrication and organoid technologies: the next frontier in hepatic and intestinal tissue engineering? Biofabrication, 9(1):013001. 10.1088/1758-5090/aa6121 PMC7116183 28211365

[115] Fatehullah A, Tan SH, Barker N (2016). Organoids as an in vitro model of human development and disease. Nat Cell Biol, 18(3):246-54. 10.1038/ncb3312 26911908

[116] Yiangou L, Ross ADB, Goh KJ, Vallier L (2018). Human Pluripotent Stem Cell-Derived Endoderm for Modeling Development and Clinical Applications. Cell Stem Cell, 22(4):485-99. 10.1016/j.stem.2018.03.016 29625066

[117] Keane TJ, Swinehart IT, Badylak SF (2015). Methods of tissue decellularization used for preparation of biologic scaffolds and in vivo relevance. Methods, 84:25-34. 10.1016/j.ymeth.2015.03.005 25791470

[118] Baptista PM, Siddiqui MM, Lozier G, Rodriguez SR, Atala A, Soker S (2011). The use of whole organ decellularization for the generation of a vascularized liver organoid. Hepatology, 53(2):604-17. 10.1002/hep.24067 21274881

[119] Jee JH, Lee DH, Ko J, Hahn S, Jeong SY, Kim HK, et al. (2019). Development of Collagen-Based 3D Matrix for Gastrointestinal Tract-Derived Organoid Culture. Stem Cells Int, 13:8472712. 10.1155/2019/8472712 PMC6595382 31312220

[120] Qin XH, Wang X, Rottmar M, Nelson BJ, Maniura-Weber K (2018). Near-Infrared Light-Sensitive Polyvinyl Alcohol Hydrogel Photoresist for Spatiotemporal Control of Cell-Instructive 3D Microenvironments. Adv Mater, 30(10):1705564. 10.1002/adma.201705564 29333748

[121] DiMarco RL, Dewi RE, Bernal G, Kuo C, Heilshorn SC (2015). Protein-engineered scaffolds for in vitro 3D culture of primary adult intestinal organoids. Biomater Sci, 3(10):1376. 10.1039/c5bm00108k PMC9063856 26371971

[122] Choi SH, Kim YH, Hebisch M, Sliwinski C, Lee S, D’Avanzo C, et al. (2014). A three-dimensional human neural cell culture model of Alzheimer’s disease. Nature, 515(7526):274-8. 10.1038/nature13800 PMC4366007 25307057

[123] Corder EH, Saunders AM, Strittmatter WJ, Schmechel DE, Gaskell PC, Small GW, et al. (1993). Gene dose of apolipoprotein E type 4 allele and the risk of Alzheimer’s disease in late onset families. Science, 261(5123):921-3. 10.1126/science.8346443 8346443

[124] Holtzman DM, Herz J, Bu G (2012). Apolipoprotein E and apolipoprotein E receptors: normal biology and roles in Alzheimer disease. Cold Spring Harb Perspect Med, 2(3):a006312. 10.1101/cshperspect.a006312 PMC3282491 22393530

[125] Mahley RW, Huang Y (1999). Apolipoprotein E: from atherosclerosis to Alzheimer’s disease and beyond. Curr Opin Lipidol, 10(3):207-17. 10.1097/00041433-199906000-00003 10431657

[126] Harris FM, Tesseur I, Brecht WJ, Xu Q, Mullendorff K, Chang S, et al. (2004). Astroglial regulation of apolipoprotein E expression in neuronal cells. Implications for Alzheimer’s disease. J Biol Chem, 279(5):3862-8. 10.1074/jbc.M309475200 14585838

[127] Koutsodendris N, Blumenfeld J, Agrawal A, Traglia M, Grone B, Zilberter M, et al. (2023). Neuronal APOE4 removal protects against tau-mediated gliosis, neurodegeneration and myelin deficits. Nat Aging, 3(3):275-96. 10.1038/s43587-023-00368-3 PMC10154214 37118426

[128] Blumenfeld J, Yip O, Kim MJ, Huang Y (2024). Cell type-specific roles of APOE4 in Alzheimer disease. Nat Rev Neurosci, 25(2):91-110. 10.1038/s41583-023-00776-9 PMC11073858 38191720

[129] Yakoub AM (2019). Cerebral organoids exhibit mature neurons and astrocytes and recapitulate electrophysiological activity of the human brain. Neural Regen Res, 14(5):757-61. 10.4103/1673-5374.249283 PMC6375034 30688257

[130] Selkoe DJ, Hardy J (2016). The amyloid hypothesis of Alzheimer’s disease at 25 years. EMBO Mol Med, 8(6):595. 10.15252/emmm.201606210 PMC4888851 27025652

[131] Sengupta A, Kabat J, Novak M, Wu Q, Grundke-Iqbal I, Iqbal K (1998). Phosphorylation of tau at both Thr 231 and Ser 262 is required for maximal inhibition of its binding to microtubules. Arch Biochem Biophys, 357(2):299-309. 10.1006/abbi.1998.0813 9735171

[132] Wang JZ, Grundke-Iqbal I, Iqbal K (2007). Kinases and phosphatases and tau sites involved in Alzheimer neurofibrillary degeneration. Eur J Neurosci, 25(1):59-68. 10.1111/j.1460-9568.2006.05226.x PMC3191918 17241267

[133] Buée L, Bussière T, Buée-Scherrer V, Delacourte A, Hof PR (2000). Tau protein isoforms, phosphorylation and role in neurodegenerative disorders. Brain Res Brain Res Rev, 33(1):95-130. 10.1016/s0165-0173(00)00019-9 10967355

[134] Iqbal K, Liu F, Gong CX (2016). Tau and neurodegenerative disease: the story so far. Nat Rev Neurol, 12(1):15-27. 10.1038/nrneurol.2015.225 26635213

[135] Shimada H, Sato Y, Sasaki T, Shimozawa A, Imaizumi K, Shindo T, et al. (2022). A next-generation iPSC-derived forebrain organoid model of tauopathy with tau fibrils by AAV-mediated gene transfer. Cell Rep Methods, 2(9):100289. 10.1016/j.crmeth.2022.100289 PMC9499998 36160042

[136] Scheltens P, Blennow K, Breteler MMB, de Strooper B, Frisoni GB, Salloway S, et al. (2016). Alzheimer’s disease. Lancet, 388(10043):505-17. 10.1016/S0140-6736(15)01124-1 26921134

[137] Pavoni S, Jarray R, Nassor F, Guyot AC, Cottin S, Rontard J, et al. (2018). Small-molecule induction of Aβ-42 peptide production in human cerebral organoids to model Alzheimer’s disease associated phenotypes. PLoS One, 13(12):e0209150. 10.1371/journal.pone.0209150 PMC6296660 30557391

[138] Cairns DM, Itzhaki RF, Kaplan DL (2022). Potential Involvement of Varicella Zoster Virus in Alzheimer’s Disease via Reactivation of Quiescent Herpes Simplex Virus Type 1. J Alzheimers Dis, 88(3):1189-200. 10.3233/JAD-220287 35754275

[139] Itzhaki RF (2023). Infections, Vaccinations, and Risk of Alzheimer’s Disease (AD)/Dementia: Probable Involvement of Reactivated Herpes Simplex Virus Type 1. Adv Exp Med Biol, 1423:279-80. 10.1007/978-3-031-31978-5_28 37525055

[140] Qiao H, Zhao W, Guo M, Zhu L, Chen T, Wang J, et al. (2022). Cerebral Organoids for Modeling of HSV-1-Induced-Amyloid β Associated Neuropathology and Phenotypic Rescue. Int J Mol Sci, 23(11):5981. 10.3390/ijms23115981 PMC9181143 35682661

[141] Alić I, Goh PA, Murray A, Portelius E, Gkanatsiou E, Gough G, et al. (2021). Patient-specific Alzheimer-like pathology in trisomy 21 cerebral organoids reveals BACE2 as a gene dose-sensitive AD suppressor in human brain. Mol Psychiatry, 26(10):5766-88. 10.1038/s41380-020-0806-5 PMC8190957 32647257

[142] Chen X, Sun G, Tian E, Zhang M, Davtyan H, Beach TG, et al. (2021). Modeling Sporadic Alzheimer’s Disease in Human Brain Organoids under Serum Exposure. Adv Sci, 8(18):e2101462. 10.1002/advs.202101462 PMC8456220 34337898

[143] Raja WK, Mungenast AE, Lin YT, Ko T, Abdurrob F, Seo J, et al. (2016). Self-Organizing 3D Human Neural Tissue Derived from Induced Pluripotent Stem Cells Recapitulate Alzheimer’s Disease Phenotypes. PLoS One, 11(9):e0161969. 10.1371/journal.pone.0161969 PMC5021368 27622770

[144] Gonzalez C, Armijo E, Bravo-Alegria J, Becerra-Calixto A, Mays CE, Soto C (2018). Modeling amyloid beta and tau pathology in human cerebral organoids. Mol Psychiatry, 23(12):2363-74. 10.1038/s41380-018-0229-8 PMC6594704 30171212

[145] Ghatak S, Dolatabadi N, Trudler D, Zhang X, Wu Y, Mohata M, et al. (2019). Mechanisms of hyperexcitability in Alzheimer’s disease hiPSC-derived neurons and cerebral organoids vs isogenic controls. Elife, 8:e50333. 10.7554/eLife.50333 PMC6905854 31782729

[146] Kuehner JN, Chen J, Bruggeman EC, Wang F, Li Y, Xu C, et al. (2021). 5-hydroxymethylcytosine is dynamically regulated during forebrain organoid development and aberrantly altered in Alzheimer’s disease. Cell Rep, 35(4):109042. 10.1016/j.celrep.2021.109042 PMC8106871 33910000

[147] Yin J, Vandongen AM (2021). Enhanced Neuronal Activity and Asynchronous Calcium Transients Revealed in a 3D Organoid Model of Alzheimer’s Disease. ACS Biomater Sci Eng, 7(1):254-64. 10.1021/acsbiomaterials.0c01583 33347288

[148] Vanova T, Sedmik J, Raska J, Amruz Cerna K, Taus P, Pospisilova V, et al. (2023). Cerebral organoids derived from patients with Alzheimer’s disease with PSEN1/2 mutations have defective tissue patterning and altered development. Cell Rep, 42(11):113310. 10.1016/j.celrep.2023.113310 37864790

[149] Holubiec MI, Alloatti M, Bianchelli J, Greloni F, Arnaiz C, Gonzalez Prinz M, et al. (2023). Mitochondrial vulnerability to oxidation in human brain organoids modelling Alzheimer’s disease. Free Radic Biol Med, 208:394-401. 10.1016/j.freeradbiomed.2023.08.028 37657763

[150] Lin YT, Seo J, Gao F, Feldman HM, Wen HL, Penney J, et al. (2018). APOE4 Causes Widespread Molecular and Cellular Alterations Associated with Alzheimer’s Disease Phenotypes in Human iPSC-Derived Brain Cell Types. Neuron, 98(6):1294. 10.1016/j.neuron.2018.06.011 PMC6048952 29953873

[151] Zhao J, Fu Y, Yamazaki Y, Ren Y, Davis MD, Liu CC, et al. (2020). APOE4 exacerbates synapse loss and neurodegeneration in Alzheimer’s disease patient iPSC-derived cerebral organoids. Nat Commun, 11(1):5540. 10.1038/s41467-020-19264-0 PMC7608683 33139712

[152] Zhao J, Ikezu TC, Lu W, Macyczko JR, Li Y, Lewis-Tuffin LJ, et al. (2023). APOE deficiency impacts neural differentiation and cholesterol biosynthesis in human iPSC-derived cerebral organoids. Stem Cell Res Ther, 14(1):214. 10.1186/s13287-023-03444-y PMC10441762 37605285

[153] Park JC, Jang SY, Lee D, Lee J, Kang U, Chang H, et al. (2021). A logical network-based drug-screening platform for Alzheimer’s disease representing pathological features of human brain organoids. Nat Commun, 12(1):280. 10.1038/s41467-020-20440-5 PMC7804132 33436582

[154] Hernández D, Rooney LA, Daniszewski M, Gulluyan L, Liang HH, Cook AL, et al. (2022). Culture Variabilities of Human iPSC-Derived Cerebral Organoids Are a Major Issue for the Modelling of Phenotypes Observed in Alzheimer’s Disease. Stem Cell Rev Rep, 18(2):718-31. 10.1007/s12015-021-10147-5 33725267

[155] Böhnke L, Traxler L, Herdy JR, Mertens J (2018). Human neurons to model aging: A dish best served old. Drug Discov Today Dis Models, 27:43-9. 10.1016/j.ddmod.2019.01.001 PMC6863511 31745399

[156] Kang E, Wang X, Tippner-Hedges R, Ma H, Folmes CDL, Gutierrez NM, et al. (2016). Age-Related Accumulation of Somatic Mitochondrial DNA Mutations in Adult-Derived Human iPSCs. Cell Stem Cell, 18(5):625-36. 10.1016/j.stem.2016.02.005 27151456

[157] Mertens J, Paquola ACM, Ku M, Hatch E, Böhnke L, Ladjevardi S, et al. (2015). Directly Reprogrammed Human Neurons Retain Aging-Associated Transcriptomic Signatures and Reveal Age-Related Nucleocytoplasmic Defects. Cell Stem Cell, 17(6):705-18. 10.1016/j.stem.2015.09.001 PMC5929130 26456686

[158] Miller JD, Ganat YM, Kishinevsky S, Bowman RL, Liu B, Tu EY, et al. (2013). Human iPSC-based modeling of late-onset disease via progerin-induced aging. Cell Stem Cell, 13(6):691-705. 10.1016/j.stem.2013.11.006 PMC4153390 24315443

[159] Tang Y, Liu ML, Zang T, Zhang CL (2017). Direct Reprogramming Rather than iPSC-Based Reprogramming Maintains Aging Hallmarks in Human Motor Neurons. Front Mol Neurosci, 10:359. 10.3389/fnmol.2017.00359 PMC5676779 29163034

[160] Arbab M BSGN (2014). Modeling motor neuron disease: the matter of time. Trends Neurosci, 37(11):642-52. 10.1016/j.tins.2014.07.008 25156326

[161] Luo C LMCRNJKJEJR (2016). Cerebral Organoids Recapitulate Epigenomic Signatures of the Human Fetal Brain. Cell Rep, 14(12):3369-84. 10.1016/j.celrep.2016.12.001 PMC5495578 28009303

[162] Patterson M, Chan DN, Ha I, Case D, Cui Y, Handel B Van, et al. (2011). Defining the nature of human pluripotent stem cell progeny. Cell Res, 22(1):178-93. 10.1038/cr.2011.133 PMC3351932 21844894

[163] Mertens J, Herdy JR, Traxler L, Schafer ST, Schlachetzki JCM, Böhnke L, et al. (2021). Age-dependent instability of mature neuronal fate in induced neurons from Alzheimer’s patients. Cell Stem Cell, 28(9):1533-1548.e6. 10.1016/j.stem.2021.04.004 PMC8423435 33910058

[164] Sun Z, Kwon JS, Ren Y, Chen S, Walker CK, Lu X, et al. (2024). Modeling late-onset Alzheimer’s disease neuropathology via direct neuronal reprogramming. Science, 385(6708):adl2992. 10.1126/science.adl2992 PMC11787906 39088624

[165] Heide M, Huttner WB, Mora-Bermúdez F (2018). Brain organoids as models to study human neocortex development and evolution. Curr Opin Cell Biol, 55:8-16. 10.1016/j.ceb.2018.06.006 30006054

[166] Quadrato G, Nguyen T, Macosko EZ, Sherwood JL, Yang SM, Berger DR, et al. (2017). Cell diversity and network dynamics in photosensitive human brain organoids. Nature, 545(7652):48-53. 10.1038/nature22047 PMC5659341 28445462

[167] Mansour AA, Gonçalves JT, Bloyd CW, Li H, Fernandes S, Quang D, et al. (2018). An in vivo model of functional and vascularized human brain organoids. Nat Biotech, 36(5):432-41. 10.1038/nbt.4127 PMC6331203 29658944

[168] Daza RAM, Englund C, Hevner RF (2007). Organotypic slice culture of embryonic brain tissue. CSH Protoc, 2007(12):4914. 10.1101/pdb.prot4914 21357004

[169] Giandomenico SL, Mierau SB, Gibbons GM, Wenger LMD, Masullo L, Sit T, et al. (2019). Cerebral organoids at the air-liquid interface generate diverse nerve tracts with functional output. Nat Neurosci, 22(4):669-79. 10.1038/s41593-019-0350-2 PMC6436729 30886407

[170] Cakir B, Xiang Y, Tanaka Y, Kural MH, Parent M, Kang YJ, et al. (2019). Engineering of human brain organoids with a functional vascular-like system. Nat Methods, 16(11):1169-75. 10.1038/s41592-019-0586-5 PMC6918722 31591580

[171] Pham MT, Pollock KM, Rose MD, Cary WA, Stewart HR, Zhou P, et al. (2018). Generation of human vascularized brain organoids. Neuroreport, 29(7):588-93. 10.1097/WNR.0000000000001014 PMC6476536 29570159

[172] Nashimoto Y, Hayashi T, Kunita I, Nakamasu A, Torisawa YS, Nakayama M, et al. (2017). Integrating perfusable vascular networks with a three-dimensional tissue in a microfluidic device. Integr Biol, 9(6):506-18. 10.1039/c7ib00024c 28561127

[173] YCOLHYZXZY Wang Y (2021). Perfused human brain organoids as a scalable platform for disease modeling and drug discovery. Stem CellS Int, 18(4):491-9.

[174] Cho CF, Wolfe JM, Fadzen CM, Calligaris D, Hornburg K, Chiocca EA, et al. (2017). Blood-brain-barrier spheroids as an in vitro screening platform for brain-penetrating agents. Nat Commun, 8(1):1-14. 10.1038/ncomms15623 PMC5467173 28585535

[175] Bergmann S, Lawler SE, Qu Y, Fadzen CM, Wolfe JM, Regan MS, et al. (2018). Blood-brain-barrier organoids for investigating the permeability of CNS therapeutics. Nat Protoc, 13(12):2827-43. 10.1038/s41596-018-0066-x PMC6673652 30382243

[176] Urich E, Patsch C, Aigner S, Graf M, Iacone R, Freskgård PO (2013). Multicellular self-assembled spheroidal model of the blood brain barrier. Sci Rep, 3:1500. 10.1038/srep01500 PMC3603320 23511305

[177] Zipser BD, Johanson CE, Gonzalez L, Berzin TM, Tavares R, Hulette CM, et al. (2007). Microvascular injury and blood-brain barrier leakage in Alzheimer’s disease. Neurobiol Aging, 28(7):977-86. 10.1016/j.neurobiolaging.2006.05.016 16782234

[178] Starr JM, Farrall AJ, Armitage P, McGurn B, Wardlaw J (2009). Blood-brain barrier permeability in Alzheimer’s disease: a case-control MRI study. Psychiatry Res, 171(3):232-41. 10.1016/j.pscychresns.2008.04.003 19211227

[179] Montagne A, Barnes SR, Sweeney MD, Halliday MR, Sagare AP, Zhao Z, et al. (2015). Blood-brain barrier breakdown in the aging human hippocampus. Neuron, 85(2):296-302. 10.1016/j.neuron.2014.12.032 PMC4350773 25611508

[180] Van De Haar HJ, Burgmans S, Jansen JFA, Van Osch MJP, Van Buchem MA, Muller M, et al. (2016). Blood-Brain Barrier Leakage in Patients with Early Alzheimer Disease. Radiology, 281(2):527-35. 10.1148/radiol.2016152244 27243267

[181] Nation DA, Sweeney MD, Montagne A, Sagare AP, D’Orazio LM, Pachicano M, et al. (2019). Blood-brain barrier breakdown is an early biomarker of human cognitive dysfunction. Nat Med, 25(2):270-6. 10.1038/s41591-018-0297-y PMC6367058 30643288

[182] Shin Y, Choi SH, Kim E, Bylykbashi E, Kim JA, Chung S, et al. (2019). Blood-Brain Barrier Dysfunction in a 3D In Vitro Model of Alzheimer’s Disease. Adv Sci, 6(20):1900962. 10.1002/advs.201900962 PMC6794630 31637161

[183] Ransohoff RM, Perry VH (2009). Microglial physiology: unique stimuli, specialized responses. Annu Rev Immunol, 27:119-45. 10.1146/annurev.immunol.021908.132528 19302036

[184] Frautschy SA, Yang F, Irrizarry M, Hyman B, Saido TC, Hsiao K, et al. (1998). Microglial response to amyloid plaques in APPsw transgenic mice. Am J Pathol, 152(1):307. PMC1858113 9422548

[185] Perlmutter LS, Barron E, Chui HC (1990). Morphologic association between microglia and senile plaque amyloid in Alzheimer’s disease. Neurosci Lett, 119(1):32-6. 10.1016/0304-3940(90)90748-x 2097581

[186] Wisniewski T, Frangione B (1992). Apolipoprotein E: a pathological chaperone protein in patients with cerebral and systemic amyloid. Neurosci Lett, 135(2):235-8. 10.1016/0304-3940(92)90444-c 1625800

[187] Frackowiak J, Wisniewski HM, Wegiel J, Merz GS, Iqbal K, Wang KC (1992). Ultrastructure of the microglia that phagocytose amyloid and the microglia that produce beta-amyloid fibrils. Acta Neuropathol, 84(3):225-33. 10.1007/BF00227813 1414275

[188] Paresce DM, Ghosh RN, Maxfield FR (1996). Microglial cells internalize aggregates of the Alzheimer’s disease amyloid beta-protein via a scavenger receptor. Neuron, 17(3):553-65. 10.1016/s0896-6273(00)80187-7 8816718

[189] Pluta R, Barcikowska M, Misicka A, Lipkowski AW, Spisacka S, Januszewski S (1999). Ischemic rats as a model in the study of the neurobiological role of human beta-amyloid peptide. Time-dependent disappearing diffuse amyloid plaques in brain. Neuroreport, 10(17):3615-9. 10.1097/00001756-199911260-00028 10619654

[190] Lee CYD, Landreth GE (2010). The role of microglia in amyloid clearance from the AD brain. J Neural Transm, 117(8):949-60. 10.1007/s00702-010-0433-4 PMC3653296 20552234

[191] Jack CR Jr, Holtzman DM (2013). Biomarker modeling of Alzheimer's disease. Neuron, 18;80(6):1347-58. 10.1016/j.neuron.2013.12.003 PMC3928967 24360540

[192] Musiek ES, Holtzman DM (2015). Three dimensions of the amyloid hypothesis: time, space and 'wingmen'. Nat Neurosci, 18(6):800-6. 10.1038/nn.4018 PMC4445458 26007213

[193] Giannakopoulos P, Herrmann FR, Bussière T, Bouras C, Kövari E, Perl DP, et al. (2003). Tangle and neuron numbers, but not amyloid load, predict cognitive status in Alzheimer's disease. Neurology, 60(9):1495-500. 10.1212/01.wnl.0000063311.58879.01 12743238

[194] Chen X, Firulyova M, Manis M, Herz J, Smirnov I, Aladyeva E, et al. (2023). Microglia-mediated T cell infiltration drives neurodegeneration in tauopathy. Nature, 615(7953):668-677. 10.1038/s41586-023-05788-0 PMC10258627 36890231

[195] Park J, Wetzel I, Marriott I, Dréau D, D’Avanzo C, Kim DY, et al. (2018). A 3D human triculture system modeling neurodegeneration and neuroinflammation in Alzheimer’s disease. Nat Neurosci, 21(7):941-51. 10.1038/s41593-018-0175-4 PMC6800152 29950669

[196] Rauch JN, & SJR (2021). TREM2 and microglia in the Alzheimer’s brain: Understanding the pathology through human brain organoid models. J Neuroinflamm, 18(1):1-13.

[197] Salameh JS, Brown RH, Berry JD (2015). Amyotrophic Lateral Sclerosis: Review. Semin Neurol, 35(4):469-76. 10.1055/s-0035-1558984 26502769

[198] Ferrer I (2018). Oligodendrogliopathy in neurodegenerative diseases with abnormal protein aggregates: The forgotten partner. Prog Neurobiol, 169:24-54. 10.1016/j.pneurobio.2018.07.004 30077775

[199] McKenzie AT, Moyon S, Wang M, Katsyv I, Song WM, Zhou X, et al. (2017). Multiscale network modeling of oligodendrocytes reveals molecular components of myelin dysregulation in Alzheimer’s disease. Mol Neurodegener, 12(1):1-20. 10.1186/s13024-017-0219-3 PMC5674813 29110684

[200] Reiche L, Küry P, Göttle P (2019). Aberrant Oligodendrogenesis in Down Syndrome: Shift in Gliogenesis? Cells, 8(12):1591. 10.3390/cells8121591 PMC6953000 31817891

[201] Wasseff SK, Scherer SS (2015). Activated immune response in an inherited leukodystrophy disease caused by the loss of oligodendrocyte gap junctions. Neurobiol Dis, 82:86-98. 10.1016/j.nbd.2015.05.018 PMC4640986 26051537

[202] Quadrato G, Brown J, Arlotta P (2016). The promises and challenges of human brain organoids as models of neuropsychiatric disease. Nat Med, 22(11):1220-8. 10.1038/nm.4214 27783065

[203] Madhavan M, Nevin ZS, Shick HE, Garrison E, Clarkson-Paredes C, Karl M, et al. (2018). Induction of myelinating oligodendrocytes in human cortical spheroids. Nat Methods, 15(9):700-706. 10.1038/s41592-018-0081-4 PMC6508550 30046099

[204] Kim H, Xu R, Padmashri R, Dunaevsky A, Liu Y, Dreyfus CF, et al. (2019). Pluripotent Stem Cell-Derived Cerebral Organoids Reveal Human Oligodendrogenesis with Dorsal and Ventral Origins. Stem Cell Rep, 12(5):890-905. 10.1016/j.stemcr.2019.04.011 PMC6524754 31091434

[205] Marton RM, Miura Y, Sloan SA, Li Q, Revah O, Levy RJ, et al. (2019) Differentiation and maturation of oligodendrocytes in human three-dimensional neural cultures. Nat Neurosci, 22(3):484-91. 10.1038/s41593-018-0316-9 PMC6788758 30692691

[206] Shaker MR, Pietrogrande G, Martin S, Lee JH, Sun W, Wolvetang EJ (2021). Rapid and Efficient Generation of Myelinating Human Oligodendrocytes in Organoids. Front Cell Neurosci, 15:631548. 10.3389/fncel.2021.631548 PMC8010307 33815061

[207] Sienski G, Narayan P, Bonner JM, Kory N, Boland S, Arczewska AA, et al. (2021). APOE4 disrupts intracellular lipid homeostasis in human iPSC-derived glia. Sci Transl Med, 13(583):eaaz4564. 10.1126/scitranslmed.aaz4564 PMC8218593 33658354

[208] Tcw J, Qian L, Pipalia NH, Chao MJ, Liang SA, Shi Y, et al. (2022). Cholesterol and matrisome pathways dysregulated in astrocytes and microglia. Cell, 185(13):2213-2233.e25. 10.1016/j.cell.2022.05.017 PMC9340815 35750033

[209] Yamazaki Y, Zhao N, Caulfield TR, Liu CC, Bu G (2019). Apolipoprotein E and Alzheimer disease: pathobiology and targeting strategies. Nat Rev Neurol, 15(9):501-18. 10.1038/s41582-019-0228-7 PMC7055192 31367008

[210] Hirschfeld LR, Risacher SL, Nho K, Saykin AJ (2022). Myelin repair in Alzheimer’s disease: a review of biological pathways and potential therapeutics. Transl Neurodegener, 11(1):1-16. 10.1186/s40035-022-00321-1 PMC9598036 36284351

[211] Mariani J, Coppola G, Zhang P, Abyzov A, Provini L, Tomasini L, et al. (2015). FOXG1-Dependent Dysregulation of GABA/Glutamate Neuron Differentiation in Autism Spectrum Disorders. Cell, 162(2):375-90. 10.1016/j.cell.2015.06.034 PMC4519016 26186191

[212] Pasca AM, Sloan SA, Clarke LE, Tian Y, Makinson CD, Huber N, et al. (2015). Functional cortical neurons and astrocytes from human pluripotent stem cells in 3D culture. Nat Methods, 12(7):671-8. 10.1038/nmeth.3415 PMC4489980 26005811

[213] Qian X, Nguyen HN, Song MM, Hadiono C, Ogden SC, Hammack C, et al. (2016). Brain-Region-Specific Organoids Using Mini-bioreactors for Modeling ZIKV Exposure. Cell, 165(5):1238-54. 10.1016/j.cell.2016.04.032 PMC4900885 27118425

[214] Hughes CS, Postovit LM, Lajoie GA (2010). Matrigel: A complex protein mixture required for optimal growth of cell culture. Proteomics, 10(9):1886-90. 10.1002/pmic.200900758 20162561

[215] McNamara NB, Munro DAD, Bestard-Cuche N, Uyeda A, Bogie JFJ, Hoffmann A, et al. (2022). Microglia regulate central nervous system myelin growth and integrity. Nature, 613(7942):120-9. 10.1038/s41586-022-05534-y PMC9812791 36517604

[216] Kong D, Park KH, Kim DH, Kim NG, Lee SE, Shin N, et al. (2023). Cortical-blood vessel assembloids exhibit Alzheimer’s disease phenotypes by activating glia after SARS-CoV-2 infection. Cell Death Discov, 9(1):32. 10.1038/s41420-022-01288-8 PMC9876421 36697403

[217] Sperling RA, LaViolette PS, O’Keefe K, O’Brien J, Rentz DM, Pihlajamaki M, et al. (2009). Amyloid deposition is associated with impaired default network function in older persons without dementia. Neuron, 63(2):178-88. 10.1016/j.neuron.2009.07.003 PMC2738994 19640477

[218] Zott B, Busche MA, Sperling RA, Konnerth A (2018). What Happens with the Circuit in Alzheimer’s Disease in Mice and Humans? Annu Rev Neurosci, 41:277-97. 10.1146/annurev-neuro-080317-061725 PMC6571139 29986165

[219] Hamm V, Héraud C, Cassei JC, Mathis C, Goutagny R (2015). Precocious Alterations of Brain Oscillatory Activity in Alzheimer’s Disease: A Window of Opportunity for Early Diagnosis and Treatment. Front Cell Neurosci, 9: 491. 10.3389/fncel.2015.00491 PMC4685112 26733816

[220] Kitchigina VF (2018). Alterations of Coherent Theta and Gamma Network Oscillations as an Early Biomarker of Temporal Lobe Epilepsy and Alzheimer’s Disease. Front Integr Neurosci, 12:36. 10.3389/fnint.2018.00036 PMC6119809 30210311

[221] Lucey BP, Holtzman DM (2015). How amyloid, sleep and memory connect. Nat Neurosci, 18(7):933. 10.1038/nn.4048 PMC4770804 26108720

[222] Braak H, Braak E (1991). Neuropathological stageing of Alzheimer-related changes. Acta Neuropathol, 82(4):239-59. 10.1007/BF00308809 1759558

[223] De Calignon A, Polydoro M, Suárez-Calvet M, William C, Adamowicz DH, Kopeikina KJ, et al. (2012). Propagation of tau pathology in a model of early Alzheimer’s disease. Neuron, 73(4):685-97. 10.1016/j.neuron.2011.11.033 PMC3292759 22365544

[224] Liu L, Drouet V, Wu JW, Witter MP, Small SA, Clelland C, et al. (2012). Trans-synaptic spread of tau pathology in vivo. PLoS One, 7(2):e31302. 10.1371/journal.pone.0031302 PMC3270029 22312444

[225] Borghese L, Dolezalova D, Opitz T, Haupt S, Leinhaas A, Steinfarz B, et al. (2010). Inhibition of notch signaling in human embryonic stem cell-derived neural stem cells delays G1/S phase transition and accelerates neuronal differentiation in vitro and in vivo. Stem Cells, 28(5):955-64. 10.1002/stem.408 20235098

